# Numerical Analysis and Comparison of Four Stabilized Finite Element Methods for the Steady Micropolar Equations

**DOI:** 10.3390/e24040454

**Published:** 2022-03-25

**Authors:** Jingnan Liu, Demin Liu

**Affiliations:** College of Mathematics and System Sciences, Xinjiang University, Urumqi 830046, China; karry13921@126.com

**Keywords:** micropolar Navier–Stokes equations, penalty method, regular method, multiscale enrichment method, local Gauss integration method, stability and convergence

## Abstract

In this paper, four stabilized methods based on the lowest equal-order finite element pair for the steady micropolar Navier–Stokes equations (MNSE) are presented, which are penalty, regular, multiscale enrichment, and local Gauss integration methods. A priori properties, existence, uniqueness, stability, and error estimation based on Fem approximation of all the methods are proven for the physical variables. Finally, some numerical examples are displayed to show the numerical characteristics of these methods.

## 1. Introduction

The MNSE is a coupling system of the conservation of mass, the conservation of linear momentum, and the conservation of angular momentum. Over the past few decades, micropolar fluids are frequently used in chemistry, physics, mechanical engineering, and medicine with the development of engineering applications. For instance, it can be used in modern lubrication theory, porous media theory, liquid crystals, suspensions, and animal blood. In this paper, let Ω be a bounded domain in $R^{n},n=2,3,$ with a sufficiently smooth boundary ∂Ω, the following MNSE in a dimensionless form will be considered:

(1) $$\left\{\begin{array}{cc} -\nu_{1}\Delta u+\nabla p=2\nu_{r}\mathrm{rot}\;\omega +f, & \mathrm{in}\;\Omega ,\\ \mathrm{div}\;u=0, & \mathrm{in}\;\Omega ,\\ -\nu_{2}\Delta \omega +4\nu_{r}\omega =2\nu_{r}\mathrm{rot}\;u+g, & \mathrm{in}\;\Omega , \end{array}\right.$$

where the fluid variables u,ω,p are the linear velocity, angular velocity, and pressure, respectively. The symbols $\nu ,\nu_{r},c_{a},c_{d}$ are used to represent some definitely given physical parameters, where $\nu_{1}=\nu +\nu_{r},\nu_{2}=c_{a}+c_{d}$ [1]. The external forces *f* and *g* are predefined. When appropriate boundary conditions are supplied to (1), the equations are well-posed. For the simplicity, we consider the following homogeneous boundary conditions:

(2) u=0,ω=0,on∂Ω.

There are many relevant results about mathematical analysis of the problem, such as the existence, uniqueness of the solution, regularity, and so on see reference [1]. In this paper, we mainly focus on numerical methods and numerical simulations of problem (1). Noting that the Galerkin variational problem of problem (1) is still a saddle-point problem, so from the viewpoint of theoretical analysis and numerical simulation, the variables, velocity, and pressure must satisfy the LBB condition either in discrete version or continuous version. The correspondingly mixed finite element spaces for these methods must be carefully chosen so that they satisfy the LBB condition. Although some stable pairs of finite elements have been studied and used widely for many years [2,3], the lowest-order finite element pairs with some supplied stabilized terms that do not meet the LBB condition also perform well. The relevant literature about stabilized mixed finite element methods for the Navier–Stokes equations are abundant, the reader can refer to [4,5,6,7] and the corresponding literature. However, the stabilized methods of the MNSE are not currently available in the literature, so it is of great significance to study these methods about the micropolar fluid.

The analysis of solutions of the MNSE has the following typical difficulties, such as incompressibility, nonlinearity, strong coupling, and multi-field coupling. Based on the above difficulties, direct numerical simulation of the MNSE will lead to a large-scale nonlinear discrete system. Therefore, it is necessary to design an efficient, accurate, and unconditionally stable numerical algorithm for the MNSE.

In this paper, the following stabilization methods, penalty method, regular method, multiscale enrichment method, and local Gauss integration method, are mainly considered [8]. Stabilized finite element methods usually have the desirable property of improving numerical stability of the standard Galerkin methods while maintaining accuracy. The stability of regular and local Gauss integration methods is achieved by introducing symmetric definite or semidefinite stabilized terms [9,10]. In [11,12,13,14], the multiscale enrichment method stabilizes the $p_{1}-p_{1}$ pair on both finite elements and their boundaries for the Stokes equations by using a multiscale approach. In [15], variational multiscale methods combined with artificial compressibility are presented for the nonstationary Navier–Stokes equations. In this paper, the above stable finite element methods will be employed for the steady micropolar flow based on the lowest order pair. Furthermore, a numerical comparison between these methods will be presented. In the case of nonlinear and nonhomogeneous boundaries, we also do some related examples, and the related theoretical results are discussed in another paper.

The rest of this paper is organized as follows: In Section 2, some notation and preliminary results for the stationary micropolar equations are introduced. Then several stabilized mixed finite element methods and their key stabilization techniques are presented. Stability and error estimates of these stabilized finite element solutions are derived in Section 3. Comparisons between these stabilized methods are performed numerically in Section 4. Finally, conclusions are stated in Section 5. Hereafter, $c,c_{i}\left(i=0,1,\ldots \right)$ is used to indicate a generic constant, which may represent different values in different situations.

## 2. Problem Statement

In this section, we introduce some notations and the well-posedness of the weak solution for continuous and discrete variational formulations of problem (1) and (2). The norm in the standard Sobolev $H^{m}\left(\Omega \right)$, m=0,1,2,…, is denoted by ${∥\cdot ∥}_{m}$. Specially, if m=0, the space $H^{0}\left(\Omega \right)$ is the general Hilbert $L^{2}\left(\Omega \right)$, endowed with $L^{2}\left(\Omega \right)$-scalar product (·,·) and norm ${∥\cdot ∥}_{0}$. The Sobolev space $H_{0}^{1}\left(\Omega \right)$ is the subspace of $H^{1}\left(\Omega \right)$ with homogeneous boundary condition. For the convenience of analysis, the following Sobolev spaces are introduced,

(3) $$\begin{array}{cc} X & =H_{0}^{1}{\left(\Omega \right)}^{n}=\left\{v\in H^{1}{\left(\Omega \right)}^{n}:v=0\;\mathrm{on}\;\partial \Omega \right\}, \end{array}$$

(4) $$\begin{array}{cc} M & =L_{0}^{2}\left(\Omega \right)=\left\{q\in L^{2}\left(\Omega \right):\int_{\Omega }q\;dx=0\right\}. \end{array}$$

From the Poincaré inequality, we know that ∀v∈X, ${∥\nabla v∥}_{0}$ and ${∥v∥}_{1}$ are equivalent norm in $H^{1}{\left(\Omega \right)}^{n}$. And $H^{-1}{\left(\Omega \right)}^{n}$ denotes the dual space of *X* with the norm:

$$\begin{array}{c} {∥f∥}_{-1}=\underset{0\neq v\in X}{\sup}\frac{\left|\langle f,v\rangle \right|}{{∥v∥}_{1}}, \end{array}$$

where 〈·,·〉 denotes duality product. Next, let introduce the following integration by parts formula for the rot operator,

(5) (rotω,u)=(ω,rotu),∀u,ω∈X.

The weak formulation for the steady micropolar Equation (1) reads: Find (u,ω,p)∈X×X×M, such that for all (v,s,q)∈X×X×M,

(6) $$\begin{array}{c} \left\{\begin{array}{c} a_{1}\left(u,v\right)-d\left(v,p\right)+d\left(u,q\right)=2\nu_{r}\left(\mathrm{rot}\;\omega ,v\right)+\left(f,v\right),\\ a_{2}\left(\omega ,s\right)+4\nu_{r}\left(\omega ,s\right)=2\nu_{r}\left(\mathrm{rot}\;u,s\right)+\left(g,s\right), \end{array}\right. \end{array}$$

where

$$\begin{array}{cc} a_{1}\left(u,v\right) & =\nu_{1}\left(\nabla u,\nabla v\right),\\ a_{2}\left(\omega ,s\right) & =\nu_{2}\left(\nabla \omega ,\nabla s\right),\\ d(v,p) & =(p,\mathrm{div}\;v). \end{array}$$

In addition, Young’s inequality will be frequently used in our analysis below:

(7) $$\begin{array}{c} ab\leq \frac{\epsilon }{p}a^{p}+\frac{\epsilon^{-\frac{p}{q}}}{q}b^{q},\;a,b,p,q,\epsilon \in R^{+},\;\frac{1}{p}+\frac{1}{q}=1,\;p,q\in \left(1,\infty \right). \end{array}$$

In order to obtain the existence and uniqueness of the weak solution of the problem (6), we should introduce the following LBB condition [2].

(8) $$\underset{q\in M}{\inf}\underset{v\in X}{\sup}\frac{d(v,q)}{∥v∥∥q∥}\geq \beta ,$$

where β>0 is a constant. Based on the general theory of saddle-point problem, the variational problem (6) is well-posed, and the following theorem holds [1,2,16]:

**Theorem** **1.** *Let $f\in L^{2}{\left(\Omega \right)}^{n},g\in L^{2}{\left(\Omega \right)}^{n}$, then there exist a unique solution (u,ω,p)∈X×X×M of the problem *(6)*, which satisfies*

(9) $$\begin{array}{c} {∥u∥}_{1}+{∥\omega ∥}_{1}+{∥p∥}_{0}\leq {c(∥f∥}_{-1}+{∥g∥}_{-1}). \end{array}$$

*Moreover, if* Ω *is regular of class $C^{2}$, then the following regularity result holds [17]:*

(10) $${∥u∥}_{2}+{∥\omega ∥}_{2}+{∥p∥}_{1}\leq {c(∥f∥}_{0}+{∥g∥}_{0}).$$

Next, we consider the discrete problem. Let $T_{h}$ be a regular triangulation of Ω made up of triangles *K*. Let $h_{K}=\mathrm{diam}\left\{K\right\},h=\max\;\left\{h_{K}:K\in T_{h}\right\}$. Associated with $T_{h}$, the finite element spaces $\left(X_{h},M_{h}\right)$ are defined:

$$\begin{array}{cc} X_{h} & =\{v\in C^{0}{\left(\bar{\Omega }\right)}^{n}\cap X:v{|}_{K}\in {\left(P_{1}\left(K\right)\right)}^{n},\forall K\in T_{h}\},\\ M_{h} & =\{q\in C^{0}\left(\bar{\Omega }\right)\cap M:q{|}_{K}\in P_{1}\left(K\right),\forall K\in T_{h}\}. \end{array}$$

Then, the Galerkin finite element formulation of the problem (6) is to seek $\left(u_{h},\omega_{h},p_{h}\right)\in \left(X_{h},X_{h},M_{h}\right)$, $\forall \left(v_{h},s_{h},q_{h}\right)\in \left(X_{h},X_{h},M_{h}\right)$ such that

(11) $$\left\{\begin{array}{c} a_{1}\left(u_{h},v_{h}\right)-d\left(v_{h},p_{h}\right)+d\left(u_{h},q_{h}\right)=2\nu_{r}\left(\mathrm{rot}\;\omega_{h},v_{h}\right)+\left(f,v_{h}\right),\\ a_{2}\left(\omega_{h},s_{h}\right)+4\nu_{r}\left(\omega_{h},s_{h}\right)=2\nu_{r}\left(\mathrm{rot}\;u_{h},s_{h}\right)+\left(g,s_{h}\right). \end{array}\right.$$

From the classical finite element theory [2,18,19], the following assumption is reasonable

**Assumption** **1.** *There exist interpolation operators $I_{h}v\in X_{h}$ such that*

(12) $$\begin{array}{cc} \; & ∥v-I_{h}{v∥}_{0}+h∥v-I_{h}{v∥}_{1}\leq ch^{2}{∥v∥}_{2}, \end{array}$$

*Furthermore, there exist the projection operator $r_{h}:M\to M_{h}$, for any given p∈M,*

(13) $$\begin{array}{c} \left(p-r_{h}p,q\right)=0,\forall q\in M_{h}, \end{array}$$

*then the following inequality holds:*

(14) $$\begin{array}{c} ∥p-r_{h}{p∥}_{0}\leq ch{∥p∥}_{1}. \end{array}$$

Now, we will consider the local interpolation operator.

(15) $$\begin{array}{c} ∥v\;-\;I_{h}{v∥}_{0,K}+h_{K}∥v\;-\;I_{h}{v∥}_{1,K}+h_{K}^{2}∥v\;-\;I_{h}{v∥}_{2,K}\leq ch_{K}^{2}{∥v∥}_{2,K},\forall v\in H^{n}\left(K\right),\\ ∥v\;-\;I_{h}{v∥}_{0,Z}+h_{Z}∥v\;-\;I_{h}{v∥}_{1,Z}\leq ch_{Z}^{\frac{3}{2}}{∥v∥}_{2,\hat{K}},\forall v\in H^{n}\left(\hat{K}\right), \end{array}$$

for $\forall K\in T_{h},Z\in \Gamma_{kj}$, where $\hat{K}=\cup \left\{K\in T_{h},Z\subset \partial K\right\}$. For pressure interpolation, we will use the Clément interpolation operator [2,20], $C_{h}:H^{1}\left(\Omega \right)\to M_{h}$ satisfying

(16) $$\begin{array}{c} ∥q\;-\;C_{h}{\left(q\right)∥}_{t,\Omega }\leq ch^{1-t}{∥q∥}_{1,\Omega },\forall q\in H^{1}\left(\Omega \right), \end{array}$$

for t=0,1.

## 3. Stabilized Mixed Element Methods

Noticing that the variational problem (6) is still a saddle-point problem, *u* and *p* are restricted by the LBB condition. In order to avoid this restriction, we introduce four stabilized methods to convert the saddle-point problem into an elliptical problem.

### 3.1. Penalty Method

The penalty method for the micropolar problem (6) is defined as: find $\left(u_{\varepsilon },\omega_{\varepsilon },p_{\varepsilon }\right)\in \left(X,X,M\right)$, such that ∀(v,s,q)∈(X,X,M),

(17) $$\left\{\begin{array}{c} a_{1}\left(u_{\varepsilon },v\right)-d\left(v,p_{\varepsilon }\right)=2\nu_{r}\left(\mathrm{rot}\;\omega_{\varepsilon },v\right)+\langle f,v\rangle ,\\ d\left(u_{\varepsilon },q\right)+\varepsilon \left(p_{\varepsilon },q\right)=0,\\ a_{2}\left(\omega_{\varepsilon },s\right)+4\nu_{r}\left(\omega_{\varepsilon },s\right)=2\nu_{r}\left(\mathrm{rot}\;u_{\varepsilon },s\right)+\langle g,s\rangle . \end{array}\right.$$

or equivalent

(18) $$\begin{array}{c} B\left(\left(u_{\varepsilon },\omega_{\varepsilon },p_{\varepsilon }\right),\left(v,s,q\right)\right)=F\left(v,s,q\right), \end{array}$$

where

(19) $$\begin{array}{cc} B(\left(u_{\varepsilon },\omega_{\varepsilon },p_{\varepsilon }\right),\left(v,s,q\right)) & =a_{1}\left(u_{\varepsilon },v\right)-d\left(v,p_{\varepsilon }\right)-2\nu_{r}\left(\mathrm{rot}\;\omega_{\varepsilon },v\right)\\ + & d\left(u_{\varepsilon },q\right)+\varepsilon \left(p_{\varepsilon },q\right)+a_{2}\left(\omega_{\varepsilon },s\right)+4\nu_{r}\left(\omega_{\varepsilon },s\right)-2\nu_{r}\left(\mathrm{rot}\;u_{\varepsilon },s\right), \end{array}$$

(20) $$\begin{array}{c} F(v,s,q)=\langle f,v\rangle +\langle g,s\rangle . \end{array}$$

**Theorem** **2.** *Suppose $f,g\in H^{-1}{\left(\Omega \right)}^{n}$, then there exists a unique solution $\left(u_{\varepsilon },\omega_{\varepsilon },p_{\varepsilon }\right)\in \left(X,X,M\right)$ of the problem* (17) *which satisfies*

(21) $$\begin{array}{c} ∥u_{\varepsilon }{∥}_{1}+\;∥\omega_{\varepsilon }{∥}_{1}+\;∥p_{\varepsilon }{∥}_{0}\;\leq {c(∥f∥}_{-1}+{∥g∥}_{-1}). \end{array}$$

**Proof.**  Obviously, $B(\left(u_{\varepsilon },\omega_{\varepsilon },p_{\varepsilon }\right),\left(v,s,q\right))$ is continuous and coercive, then by using the Lax–Milgram theorem, we get the existence and uniqueness of solution. Next, taking $v=u_{\varepsilon },s=\omega_{\varepsilon },q=p_{\varepsilon }$ in problem (17) to get

$$\begin{array}{cc} \; & \nu_{1}∥u_{\varepsilon }{∥}_{1}^{2}+\;\nu_{2}∥\omega_{\varepsilon }{∥}_{1}^{2}+\;4\nu_{r}∥\omega_{\varepsilon }{∥}_{0}^{2}-\;4\nu_{r}∥u_{\varepsilon }{∥}_{1}∥\omega_{\varepsilon }{∥}_{0}+\;\varepsilon {∥p_{\varepsilon }∥}_{0}^{2}\\ \; & \geq \nu ∥u_{\varepsilon }{∥}_{1}^{2}+\;\nu_{2}∥\omega_{\varepsilon }{∥}_{1}^{2}\;\geq \min\left\{\nu ,\nu_{2}\right\}(∥u_{\varepsilon }{∥}_{1}^{2}+\;∥\omega_{\varepsilon }{∥}_{1}^{2}). \end{array}$$

(22) $$\begin{array}{c} \nu_{1}∥u_{\varepsilon }{∥}_{1}^{2}+\;\varepsilon ∥p_{\varepsilon }{∥}_{0}^{2}\;\leq 2\nu_{r}∥u_{\varepsilon }{∥}_{1}∥\omega_{\varepsilon }{∥}_{0}+\;{∥f∥}_{-1}{∥u_{\varepsilon }∥}_{1}, \end{array}$$

(23) $$\begin{array}{c} \nu_{2}∥\omega_{\varepsilon }{∥}_{1}^{2}+\;4\nu_{r}∥\omega_{\varepsilon }{∥}_{0}^{2}\;\leq 2\nu_{r}∥u_{\varepsilon }{∥}_{1}∥\omega_{\varepsilon }{∥}_{0}+\;{∥g∥}_{-1}{∥\omega_{\varepsilon }∥}_{1}. \end{array}$$

Adding (22) and (23), we get

$$\begin{array}{cc} \; & \min\left\{\nu ,\nu_{2}\right\}(∥u_{\varepsilon }{∥}_{1}^{2}+\;∥\omega_{\varepsilon }{∥}_{1}^{2})\;+\;\varepsilon ∥p_{\varepsilon }{∥}_{0}^{2}\;\leq {∥f∥}_{-1}∥u_{\varepsilon }{∥}_{1}+\;{∥g∥}_{-1}{∥\omega_{\varepsilon }∥}_{1}\\ \leq & \frac{1}{2}\min\left\{\nu ,\nu_{2}\right\}∥u_{\varepsilon }{∥}_{1}^{2}+\;\frac{1}{2}\min{\left\{\nu ,\nu_{2}\right\}}^{-1}{∥f∥}_{-1}^{2}+\frac{1}{2}\min\left\{\nu ,\nu_{2}\right\}∥\omega_{\varepsilon }{∥}_{1}^{2}+\;\frac{1}{2}\min{\left\{\nu ,\nu_{2}\right\}}^{-1}{∥g∥}_{-1}^{2}, \end{array}$$

thus, we obtain

$$\begin{array}{c} \min\left\{\nu ,\nu_{2}\right\}(∥u_{\varepsilon }{∥}_{1}^{2}+\;∥\omega_{\varepsilon }{∥}_{1}^{2})\;+\;2\varepsilon ∥p_{\varepsilon }{∥}_{0}^{2}\;\leq \min{\left\{\nu ,\nu_{2}\right\}}^{-1}{(∥f∥}_{-1}^{2}+{∥g∥}_{-1}^{2}). \end{array}$$

Then,

$$\begin{array}{c} ∥u_{\varepsilon }{∥}_{1}+\;∥\omega_{\varepsilon }{∥}_{1}\;\leq \min{\left\{\nu ,\nu_{2}\right\}}^{-\frac{1}{2}}{(∥f∥}_{-1}+{∥g∥}_{-1}). \end{array}$$

On the other hand, by the LBB condition, taking q=0 in (17),

$$\begin{array}{c} \beta ∥p_{\varepsilon }{∥}_{0}\;\leq \max\left\{\nu_{1},2\nu_{r}\right\}(∥u_{\varepsilon }{∥}_{1}+\;∥\omega_{\varepsilon }{∥}_{1}{)\;+∥f∥}_{-1}\leq {c(∥f∥}_{-1}+{∥g∥}_{-1}). \end{array}$$

□

Let consider the relationship between the penalty problem and the solution of the variational problem.

**Theorem** **3.** *Suppose (u,ω,p), $\left(u_{\varepsilon },\omega_{\varepsilon },p_{\varepsilon }\right)$ are the solutions of* (6) *and* (17) *respectively, then there exist*

(24) $$\begin{array}{c} ∥u\;-\;u_{\varepsilon }{∥}_{1}+∥\omega \;-\;\omega_{\varepsilon }{∥}_{1}+\;{∥p-p_{\varepsilon }∥}_{0}\leq c\sqrt{\varepsilon }. \end{array}$$

**Proof.**  Subtracting (17) from (11), we get

(25) $$\begin{array}{c} a_{1}\left(u-u_{\varepsilon },v\right)-d\left(v,p-p_{\varepsilon }\right)+d\left(u-u_{\varepsilon },q\right)-\varepsilon \left(p_{\varepsilon },q\right)=2\nu_{r}\left(\mathrm{rot}\;\left(\omega -\omega_{\varepsilon }\right),v\right),\\ a_{2}\left(\omega -\omega_{\varepsilon },s\right)+4\nu_{r}\left(\omega -\omega_{\varepsilon },s\right)=2\nu_{r}\left(\mathrm{rot}\;\left(u-u_{\varepsilon }\right),s\right). \end{array}$$

Taking $v=u-u_{\varepsilon }$, $s=\omega -\omega_{\varepsilon }$, $q=p-p_{\varepsilon }$ in (25),

(26) $$\begin{array}{cc} \; & a_{1}\left(u-u_{\varepsilon },u-u_{\varepsilon }\right)+a_{2}\left(\omega -\omega_{\varepsilon },\omega -\omega_{\varepsilon }\right)+4\nu_{r}\left(\omega -\omega_{\varepsilon },\omega -\omega_{\varepsilon }\right)\\ \; & -4\nu_{r}\left(\omega -\omega_{\varepsilon },\mathrm{rot}\;\left(u-u_{\varepsilon }\right)\right)-\varepsilon \left(p_{\varepsilon },p-p_{\varepsilon }\right)=0, \end{array}$$

Furthermore, we have

$$\begin{array}{cc} \; & a_{1}\left(u-u_{\varepsilon },u-u_{\varepsilon }\right)+a_{2}\left(\omega -\omega_{\varepsilon },\omega -\omega_{\varepsilon }\right)+4\nu_{r}\left(\omega -\omega_{\varepsilon },\omega -\omega_{\varepsilon }\right)\\ \; & -4\nu_{r}\left(\omega -\omega_{\varepsilon },\mathrm{rot}\;\left(u-u_{\varepsilon }\right)\right)+\varepsilon \left(p_{\varepsilon },p_{\varepsilon }\right)=\varepsilon \left(p_{\varepsilon },p\right), \end{array}$$

Thus

$$\begin{array}{c} \nu ∥u\;-\;u_{\varepsilon }{∥}_{1}^{2}+\;\nu_{2}∥\omega \;-\;\omega_{\varepsilon }{∥}_{1}^{2}\;\leq \frac{\varepsilon }{2}{∥p∥}_{0}^{2}+\frac{\varepsilon }{2}{∥p_{\varepsilon }∥}_{0}^{2}. \end{array}$$

By using (9) and (21),

$$\begin{array}{c} \nu ∥u\;-\;u_{\varepsilon }{∥}_{1}^{2}+\;\nu_{2}∥\omega \;-\;\omega_{\varepsilon }{\;∥}_{1}^{2}\leq {\varepsilon (∥f∥}_{-1}+{∥g∥}_{-1}{)}^{2}. \end{array}$$

On the other hand, taking q=0 in (25). For the penalty method, d(v,q) still satisfies the LBB conditions, an estimate of the pressure can be obtained

$$\begin{array}{cc} \beta ∥p\;-\;p_{\epsilon }{∥}_{0} & \leq \underset{v\in X}{\sup}\frac{\left(\nabla \cdot v,∥p-p_{\epsilon }∥\right)}{{∥v∥}_{1}}\\ \; & \leq \underset{v\in X}{\sup}\frac{|a_{1}\left(u-u_{\varepsilon },v\right)|\;+\;|2\nu_{r}\left(\mathrm{rot}\;\left(\omega -\omega_{\varepsilon }\right),v\right)|}{{∥v∥}_{1}}\\ \; & \leq \nu_{1}∥u\;-\;u_{\epsilon }{∥}_{1}+\;2\sqrt{2}\nu_{r}{∥\omega \;-\;\omega_{\varepsilon }∥}_{1}\\ \; & \leq \max\left\{\nu_{1},2\sqrt{2}\nu_{r}\right\}(∥u-u_{\epsilon }{∥}_{1}+∥\omega \;-\;\omega_{\varepsilon }{∥}_{1}), \end{array}$$

which implies that

(27) $$\begin{array}{c} ∥p\;-\;p_{\epsilon }{∥}_{0}\;\leq \frac{\max\{\nu_{1},2\sqrt{2}\nu_{r}\}}{\beta }(∥u-u_{\epsilon }{∥}_{1}+∥\omega \;-\;\omega_{\varepsilon }{∥}_{1}). \end{array}$$

□

Next, let consider the corresponding discrete weak form of the penalty method (17): find $\left(u_{\varepsilon h},\omega_{\varepsilon h},p_{\varepsilon h}\right)\in \left(X_{h},X_{h},M_{h}\right)$ such that for all $\left(v,q,s\right)\in \left(X_{h},X_{h},M_{h}\right)$,

(28) $$\left\{\begin{array}{c} a_{1}\left(u_{\varepsilon h},v\right)-d\left(v,p_{\varepsilon h}\right)+d\left(u_{\varepsilon h},q\right)+\varepsilon \left(p_{\varepsilon h},q\right)=2\nu_{r}\left(\mathrm{rot}\;\omega_{\varepsilon h},v\right)+\left(f,v\right),\\ a_{2}\left(\omega_{\varepsilon h},s\right)+4\nu_{r}\left(\omega_{\varepsilon h},s\right)=2\nu_{r}\left(\mathrm{rot}\;u_{\varepsilon h},s\right)+\left(g,s\right). \end{array}\right.$$

**Theorem** **4.** *The problem* (28) *exists a unique solution $\left(u_{\varepsilon h},\omega_{\varepsilon h},p_{\varepsilon h}\right)\in \left(X_{h},W_{h},M_{h}\right)$ such that*

(29) $$\begin{array}{c} ∥u_{\varepsilon h}{∥}_{1}+\;∥\omega_{\varepsilon h}{∥}_{1}+\;\sqrt{\varepsilon }∥p_{\varepsilon h}{∥}_{0}\;\leq {c(∥f∥}_{-1}+{∥g∥}_{-1}). \end{array}$$

**Proof.**  Choosing $\left(v,s,q\right)=\left(u_{\varepsilon h},\omega_{\varepsilon h},p_{\varepsilon h}\right)$ in (28), we obtain

$$\begin{array}{cc} \; & \nu_{1}∥u_{\varepsilon h}{∥}_{1}^{2}+\;\nu_{2}∥\omega_{\varepsilon h}{∥}_{1}^{2}+\;\varepsilon ∥p_{\varepsilon h}{∥}_{0}^{2}+\;4\nu_{r}{∥\omega_{\varepsilon h}∥}_{0}^{2}\\ \; & \leq 4\nu_{r}∥u_{\varepsilon h}{∥}_{1}∥\omega_{\varepsilon h}{∥}_{0}+\;{∥f∥}_{-1}∥u_{\varepsilon h}{∥}_{1}+\;{∥g∥}_{-1}{∥\omega_{\varepsilon h}∥}_{1}. \end{array}$$

By using Young’s inequality (7), we have

$$\begin{array}{cc} 4\nu_{r}∥u_{\varepsilon h}{∥}_{1}{∥\omega_{\varepsilon h}∥}_{0}\leq & \nu_{r}∥u_{\varepsilon h}{∥}_{1}^{2}+\;4\nu_{r}{∥\omega_{\varepsilon h}∥}_{0}^{2},\\ {∥f∥}_{-1}{∥u_{\varepsilon h}∥}_{1}\leq & \frac{1}{2}\min\;{\left\{\nu ,\nu_{2}\right\}}^{-1}{∥f∥}_{-1}^{2}+\frac{1}{2}\min\;\left\{\nu ,\nu_{2}\right\}{∥u_{\varepsilon h}∥}_{1}^{2},\\ {∥g∥}_{-1}{∥\omega_{\varepsilon h}∥}_{1}\leq & \frac{1}{2}\min\;{\left\{\nu ,\nu_{2}\right\}}^{-1}{∥g∥}_{-1}^{2}+\frac{1}{2}\min\;\left\{\nu ,\nu_{2}\right\}{∥\omega_{\varepsilon h}∥}_{1}^{2}, \end{array}$$

Then, carrying the above inequalities to get

(30) $$\begin{array}{c} \nu ∥u_{\varepsilon h}{∥}_{1}^{2}+\;\nu_{2}∥\omega_{\varepsilon h}{∥}_{1}^{2}+\;\varepsilon ∥p_{\varepsilon h}{∥}_{0}^{2}\;\leq {∥f∥}_{-1}∥u_{\varepsilon h}{∥}_{1}+\;{∥g∥}_{-1}{∥\omega_{\varepsilon h}∥}_{1}, \end{array}$$

and hence,

$$\begin{array}{cc} \; & \min\;\left\{\nu ,\nu_{2}\right\}(∥u_{\varepsilon h}{∥}_{1}^{2}+\;∥\omega_{\varepsilon h}{∥}_{1}^{2})\;+\;\varepsilon ∥p_{\varepsilon h}{∥}_{0}^{2}\\ \leq & \frac{1}{2}\min\;{\left\{\nu ,\nu_{2}\right\}}^{-1}{∥f∥}_{-1}^{2}+\frac{1}{2}\min\;\left\{\nu ,\nu_{2}\right\}∥u_{\varepsilon h}{∥}_{1}^{2}+\;\frac{1}{2}\min\;{\left\{\nu ,\nu_{2}\right\}}^{-1}{∥g∥}_{-1}^{2}\\ \; & +\frac{1}{2}\min\;\left\{\nu ,\nu_{2}\right\}{∥\omega_{\varepsilon h}∥}_{1}^{2}, \end{array}$$

thus,

$$\begin{array}{c} \min\;\left\{\nu ,\nu_{2}\right\}(∥u_{\varepsilon h}{∥}_{1}^{2}+\;∥\omega_{\varepsilon h}{∥}_{1}^{2})\;+\;2\varepsilon ∥p_{\varepsilon h}{∥}_{0}^{2}\;\leq \min\;{\left\{\nu ,\nu_{2}\right\}}^{-1}{(∥f∥}_{-1}^{2}+{∥g∥}_{-1}^{2}), \end{array}$$

the above inequality implies that

$$\begin{array}{cc} \min\;\left\{\nu ,\nu_{2}\right\}{∥u_{\varepsilon h}∥}_{1}^{2}\leq & \min\;{\left\{\nu ,\nu_{2}\right\}}^{-1}{(∥f∥}_{-1}^{2}+{∥g∥}_{-1}^{2}),\\ ∥u_{\varepsilon h}{∥}_{1}\leq & \min\;{\left\{\nu ,\nu_{2}\right\}}^{-1}{(∥f∥}_{-1}+{∥g∥}_{-1});\\ \min\;\left\{\nu ,\nu_{2}\right\}{∥\omega_{\varepsilon h}∥}_{1}^{2}\leq & \min\;{\left\{\nu ,\nu_{2}\right\}}^{-1}{(∥f∥}_{-1}^{2}+{∥g∥}_{-1}^{2}),\\ ∥\omega_{\varepsilon h}{∥}_{1}\leq & \min\;{\left\{\nu ,\nu_{2}\right\}}^{-1}{(∥f∥}_{-1}+{∥g∥}_{-1}). \end{array}$$

On the other hand, we obtain from (30)

$$\begin{array}{c} \sqrt{\varepsilon }∥p_{\varepsilon h}{∥}_{0}\;\leq {c(∥f∥}_{-1}+{∥g∥}_{-1}). \end{array}$$

□

**Theorem** **5.** *Let $\left(u_{\varepsilon },\omega_{\varepsilon },p_{\varepsilon }\right)$ and $\left(u_{\varepsilon h},p_{\varepsilon h},\omega_{\varepsilon h}\right)$ be the solution of *(17) *and* (28)*, respectively. Then the error satisfies*

(31) $$∥u_{\varepsilon }-u_{\varepsilon h}{∥}_{1}+\;∥\omega_{\varepsilon }-\omega_{\varepsilon h}{∥}_{1}+\;\sqrt{\varepsilon }{∥p_{\varepsilon }-p_{\varepsilon h}∥}_{0}\leq c\left(h+\frac{h}{\sqrt{\varepsilon }}\right),$$

**Proof.**  Subtracting (28) from (17) yields

(32) $$\begin{array}{cc} a_{1}\left(u_{\varepsilon }-u_{\varepsilon h},v_{h}\right)-d\left(v_{h},p_{\varepsilon }-p_{\varepsilon h}\right)+d\left(u_{\varepsilon }-u_{\varepsilon h},q\right)+\varepsilon \left(p_{\varepsilon }-p_{\varepsilon h},q\right)= & 2\nu_{r}\left(\mathrm{rot}\;\left(\omega_{\varepsilon }-\omega_{\varepsilon h}\right),v_{h}\right),\\ a_{2}\left(\omega_{\varepsilon }-\omega_{\varepsilon h},s_{h}\right)+4\nu_{r}\left(\left(\omega_{\varepsilon }-\omega_{\varepsilon h}\right),s\right)= & 2\nu_{r}\left(\mathrm{rot}\;\left(\omega_{\varepsilon }-\omega_{\varepsilon h}\right),s_{h}\right). \end{array}$$

Denoting $\left(e,\theta ,\eta \right)=\left(I_{h}u_{\varepsilon }-u_{\varepsilon h},I_{h}\omega_{\varepsilon }-\omega_{\varepsilon h},r_{h}p_{\varepsilon }-p_{\varepsilon h}\right)$ with (e,θ,η)=(v,s,q), we have

$$\begin{array}{cc} \nu_{1}{∥e∥}_{1}^{2}+\varepsilon {∥\eta ∥}_{0}^{2}\leq & |\nu_{1}\left(\nabla \left(u_{\varepsilon }-I_{h}u_{\varepsilon }\right),\nabla e\right)|\;+\;2\nu_{r}\left(\mathrm{rot}\;\theta ,e\right)+\left|2\nu_{r}\left(\mathrm{rot}\;\left(\omega_{\varepsilon }-I_{h}\omega_{\varepsilon }\right),e\right)\right|\\ \; & +\;|d\left(e,p_{\varepsilon }-r_{h}p_{\varepsilon h}\right)|\;+\;|d\left(u_{\varepsilon }-I_{h}u_{\varepsilon h},\eta \right)\left|\;+\;\varepsilon \right|\left(p_{\varepsilon }-r_{h}p_{\varepsilon },\eta \right)|,\\ \nu_{2}{∥\theta ∥}_{1}^{2}+4\nu_{r}{∥\theta ∥}_{0}^{2}\leq & |\nu_{2}\left(\nabla \left(\omega_{\varepsilon }-I_{h}\omega_{\varepsilon }\right),\theta \right)|\;+\;|4\nu_{r}\left(\omega_{\varepsilon }-I_{h}\omega_{\varepsilon }\right),\theta )|+2\nu_{r}\left(\mathrm{rot}\;e,\theta \right)\\ \; & +\;2\nu_{r}\left(\mathrm{rot}\;\left(u_{\varepsilon }-I_{h}u_{\varepsilon }\right),\theta \right). \end{array}$$

Adding the above two formulas together and simplifying, we get

$$\begin{array}{cc} {\nu ∥e∥}_{1}^{2}+\nu_{2}{∥\theta ∥}_{1}^{2}+\varepsilon {∥\eta ∥}_{0}^{2}\leq & |\nu_{1}\left(\nabla \left(u_{\varepsilon }-I_{h}u_{\varepsilon }\right),\nabla e\right)\left|\;+\;\right|2\nu_{r}\left(\mathrm{rot}\;\left(\omega_{\varepsilon }-I_{h}\omega_{\varepsilon }\right),e\right)+d\left(e,p_{\varepsilon }-r_{h}p_{\varepsilon h}\right)\\ \; & +\;|d\left(u_{\varepsilon }-I_{h}u_{\varepsilon h},\eta \right)\left|\;+\;\varepsilon \right|\left(p_{\varepsilon }-r_{h}p_{\varepsilon },\eta \right)|\;+\;\nu_{2}\left|\left(\nabla \left(\omega_{\varepsilon }-I_{h}\omega_{\varepsilon }\right),\theta \right)\right|\\ \; & +\;4\nu_{r}\left(\mathrm{rot}\;e,\theta \right)+2\nu_{r}\left(\mathrm{rot}\;\left(u_{\varepsilon }-I_{h}u_{\varepsilon }\right),\theta \right)\\ \leq & {ch(∥e∥}_{1}+{∥\theta ∥}_{1}+{ch∥\eta ∥}_{0})(∥u_{\varepsilon }-I_{h}u_{\varepsilon }{∥}_{1}+\;∥\omega_{\varepsilon }-I_{h}\omega_{\varepsilon }{∥}_{1}+\;∥p_{\varepsilon }-r_{h}p_{\varepsilon }{∥}_{0}), \end{array}$$

Now, by using Hölder and Young’s inequalities, we obtain

$$\begin{array}{c} {∥e∥}_{1}+{∥\theta ∥}_{1}+\sqrt{\varepsilon }{∥\eta ∥}_{0}\leq c\frac{h}{\sqrt{\varepsilon }}. \end{array}$$

Applying the triangle inequality to gain

$$\begin{array}{c} ∥u_{\varepsilon }-u_{\varepsilon h}{∥}_{1}+\;∥\omega_{\varepsilon }-\omega_{\varepsilon h}{∥}_{1}+\;\sqrt{\varepsilon }{∥p_{\varepsilon }-p_{\varepsilon h}∥}_{0}\leq c\left(h+\frac{h}{\sqrt{\varepsilon }}\right). \end{array}$$

□

**Theorem** **6.** *Under the Theorems 3 and 5, the errors $∥u\;-\;u_{\varepsilon h}∥,∥\omega -\omega_{\varepsilon h}∥$ and $∥p\;-\;p_{\varepsilon h}∥$ satisfy*

$$\begin{array}{c} ∥u\;-\;u_{\varepsilon h}{∥}_{1}+∥\omega \;-\;\omega_{\varepsilon h}{∥}_{1}+\;\sqrt{\varepsilon }{∥p-p_{\varepsilon h}∥}_{0}\leq c\left(\varepsilon +h+\frac{h}{\sqrt{\varepsilon }}\right). \end{array}$$

**Proof.**  The result based on (24) and (31), we get the above inequality. □

### 3.2. Regular Method

The variational problem (6) of the regular method is: find $\left(u_{Rh},\omega_{Rh},p_{Rh}\right)\in \left(X_{h},X_{h},M_{h}\right)$ such that

(33) $$\begin{array}{c} B_{2}\left(\left(u_{Rh},\omega_{Rh},p_{Rh}\right),\left(v,s,q\right)\right)=F\left(v,s,q\right),\;\forall \left(v,s,q\right)\in \left(X_{h},X_{h},M_{h}\right), \end{array}$$

where

(34) $$\begin{array}{cc} B_{2}\left(\left(u_{Rh},\omega_{Rh},p_{Rh}\right),\left(v,s,q\right)\right)=\; & a_{1}\left(u_{Rh},v\right)+a_{2}\left(\omega_{Rh},s\right)+4\nu_{r}\left(\omega_{Rh},s\right)-\left(\mathrm{div}\;v,p_{Rh}\right)\\ \; & +\left(\mathrm{div}\;u_{Rh},q\right)-2\nu_{r}\left(\mathrm{rot}\;\omega_{Rh},v\right)-2\nu_{r}\left(\mathrm{rot}\;u_{Rh},s\right)\\ \; & +\tau_{K}\sum_{K\in T_{h}}{\left(\nabla p_{Rh}-2\nu_{r}\mathrm{rot}\;\omega_{Rh},\nabla q\right)}_{K}. \end{array}$$

$$\begin{array}{cc} F(v,s,q) & =\left(f,v\right)+\left(g,s\right)+\sum_{K\in T_{h}}\left(f,\nabla q\right), \end{array}$$

where the stabilized parameter $\tau_{K}=\beta_{K}\frac{h_{K}^{2}}{\nu_{1}}$. For the pressure variable p∈M, we define the mesh-dependent norm

(35) $${∥p∥}_{h}={\left\{\tau_{K}\sum_{K\in T_{h}}{∥p∥}_{1,K}^{2}\right\}}^{\frac{1}{2}}.$$

Next, let prove the following continuous and elliptical properties for bilinear form $B_{2}\left(\left(u_{Gh},\omega_{Gh},p_{Gh}\right),\left(v,s,q\right)\right)$.

**Lemma** **1.** *The bilinear form $B_{2}\left(\left(u_{Rh},\omega_{Rh},p_{Rh}\right),\left(v,s,q\right)\right)$, $\forall \left(v,s,q\right)\in X_{h}\times W_{h}\times M_{h}$ satisfies the continuous property:*

(36) $$\begin{array}{c} B_{2}\left(\left(u_{Rh},\omega_{Rh},p_{Rh}\right),\left(v,s,q\right)\right)\leq c(∥u_{Rh}{∥}_{1}+\;∥\omega_{Rh}{∥}_{1}+\;∥p_{Rh}{∥}_{0}{)(∥v∥}_{1}+{∥s∥}_{1}+{∥q∥}_{0}). \end{array}$$

*and the elliptical property:*

(37) $$\begin{array}{cc} \; & B_{2}\left(\left(u_{Rh},\omega_{Rh},p_{Rh}\right),\left(u_{Rh},\omega_{Rh},p_{Rh}\right)\right)\geq c(∥u_{Rh}{∥}_{1,K}^{2}+\;∥\omega_{Rh}{∥}_{1,K}^{2}+\;∥p_{Rh}{∥}_{h}^{2}). \end{array}$$

*$\forall \left(u_{Rh},\omega_{Rh},p_{Rh}\right)\in X_{h}\times W_{h}\times M_{h}$.*

**Proof.**  The continuous property is obvious. Let first consider the restriction of $B_{2}\left(\cdot ,\cdot \right)$ on each element *K*, by using Schwarz’s inequality,

(38) $$\begin{array}{cc} \; & B_{2}{\left(\left(u_{Rh},\omega_{Rh},p_{Rh}\right),\left(u_{Rh},\omega_{Rh},p_{Rh}\right)\right)}_{K}\\ = & \nu_{1}∥u_{Rh}{∥}_{1,K}^{2}+\;\nu_{2}∥\omega_{Rh}{∥}_{1,K}^{2}+\;4\nu_{r}∥\omega_{Rh}{∥}_{0,K}^{2}+\;\tau_{K}{∥p_{Rh}∥}_{1,K}^{2}\\ \; & -\;2\nu_{r}{\left(\mathrm{rot}\;\omega_{Rh},u_{Rh}\right)}_{K}-2\nu_{r}{\left(\mathrm{rot}\;u_{Rh},\omega_{Rh}\right)}_{K}\\ \geq & \nu_{1}∥u_{Rh}{∥}_{1,K}^{2}+\;\nu_{2}∥\omega_{Rh}{∥}_{1,K}^{2}+\;4\nu_{r}∥\omega_{Rh}{∥}_{0,K}^{2}+\;\tau_{K}{∥p_{Rh}∥}_{1,K}^{2}\\ \; & -\;4\nu_{r}{\left(\omega_{Rh},\mathrm{rot}\;u_{Rh}\right)}_{K}. \end{array}$$

Applying Young’s inequality with $\epsilon =\frac{1}{2}$, we can get the following estimate of (38)

$$\begin{array}{cc} \; & B_{2}{\left(\left(u_{Rh},\omega_{Rh},p_{Rh}\right),\left(u_{Rh},\omega_{Rh},p_{Rh}\right)\right)}_{K}\\ \geq & \left(\nu_{1}-2\nu_{r}\epsilon \right)∥u_{Rh}{∥}_{1,K}^{2}+\;\nu_{2}∥\omega_{Rh}{∥}_{1,K}^{2}+\left(4\nu_{r}-\frac{2\nu_{r}}{\epsilon }\right)∥\omega_{Rh}{∥}_{0,K}^{2}+\;\tau_{K}{∥p_{Rh}∥}_{1,K}^{2}\\ \geq & \min\;\left\{\nu ,\nu_{2}\right\}(∥u_{Rh}{∥}_{1,K}^{2}+\;∥\omega_{Rh}{∥}_{1,K}^{2})\;+\;\tau_{K}{∥p_{Rh}∥}_{1,K}^{2}, \end{array}$$

on each *K*, and the proof is finished by adding $K\in T_{h}$. □

We introduce the following approximation properties [4].

**Lemma** **2.** *Let $\left(v,s,q\right)\in {\left[H^{2}\left(\Omega \right)\cap H_{0}^{1}\left(\Omega \right)\right]}^{n}\times {\left[H^{2}\left(\Omega \right)\cap H_{0}^{1}\left(\Omega \right)\right]}^{n}\times \left[H^{1}\left(\Omega \right)\cap L_{0}^{2}\left(\Omega \right)\right]$, there exists an interpolation $I_{h}v$ such that*

(39) $$\begin{array}{c} ||v\;-\;I_{h}{v||}_{1}^{2}+\sum_{K\in T_{h}}\tau_{K}^{-1}∥v\;-\;I_{h}{v∥}_{0,K}^{2}\leq ch^{2}{∥v∥}_{2,\Omega }^{2}. \end{array}$$

**Theorem** **7.** *Let $\left(u,\omega ,p\right)\in {\left[H^{2}\left(\Omega \right)\cap H_{0}^{1}\left(\Omega \right)\right]}^{n}\times {\left[H^{2}\left(\Omega \right)\cap H_{0}^{1}\left(\Omega \right)\right]}^{n}\times \left[H^{1}\left(\Omega \right)\cap L_{0}^{2}\left(\Omega \right)\right]$ be the solution of *(1)*, then the finite element solution $\left(u_{Rh},\omega_{Rh},p_{Rh}\right)$ to *(33) *satisfies the following error estimate*

(40) $$\begin{array}{c} ∥u\;-\;u_{Rh}{∥}_{1,\Omega }+∥\omega \;-\;\omega_{Rh}{∥}_{1,\Omega }+∥p\;-\;p_{Rh}{∥}_{h}\;\leq {ch(∥u∥}_{2,\Omega }+{∥\omega ∥}_{2,\Omega }+{∥p∥}_{1,\Omega }) \end{array}$$

**Proof.**  Let

(41) $$\begin{array}{cccccc} e & =u_{Rh}-I_{h}u, & \eta & =p_{Rh}-r_{h}p, & \theta & =\omega_{Rh}-I_{h}\omega ,\\ e_{h} & =u-I_{h}u, & \eta_{h} & =p-r_{h}p, & \theta_{h} & =\omega -I_{h}\omega , \end{array}$$

Then, from Lemma 1, using the definition of $B_{2}$, we obtain

$$\begin{array}{cc} \; & B_{2}(\left(e,\theta ,\eta \right),\left(e,\theta ,\eta \right)=B_{2}\left(\left(e_{h},\theta_{h},\eta_{h}\right),\left(e,\theta ,\eta \right)\right)\\ = & \nu_{1}{\left(\nabla e_{h},\nabla e\right)}_{\Omega }+\nu_{2}{\left(\nabla \theta_{h},\nabla \theta \right)}_{\Omega }+4\nu_{r}{\left(\theta_{h},\theta \right)}_{\Omega }-{\left(\mathrm{div}\;e,\eta_{h}\right)}_{\Omega }\\ \; & +\;{\left(\mathrm{div}\;e_{h},\eta \right)}_{\Omega }-2\nu_{r}{\left(\mathrm{rot}\;\theta_{h},e\right)}_{\Omega }-2\nu_{r}{\left(\mathrm{rot}\;e_{h},\theta \right)}_{\Omega }\\ \; & +\;\tau_{K}\sum_{K\in T_{h}}{\left(\nabla \eta_{h}-2\nu_{r}\mathrm{rot}\;\theta_{h},\nabla \eta \right)}_{K}, \end{array}$$

but, since $e_{h}$ vanishes on ∂Ω (since *u* belongs to $H_{0}^{1}{\left(\Omega \right)}^{n}$), after integration by parts

$$\begin{array}{cc} \; & B_{2}(\left(e,\theta ,\eta \right),\left(e,\theta ,\eta \right)\\ = & \nu_{1}{\left(\nabla e_{h},\nabla e\right)}_{\Omega }+\nu_{2}{\left(\nabla \theta_{h},\nabla \theta \right)}_{\Omega }+4\nu_{r}{\left(\theta_{h},\theta \right)}_{\Omega }-2\nu_{r}{\left(\mathrm{rot}\;\theta_{h},e\right)}_{\Omega }-2\nu_{r}{\left(\mathrm{rot}\;e_{h},\theta \right)}_{\Omega }\\ \; & -\;{\left(\mathrm{div}\;e,\eta_{h}\right)}_{\Omega }-{\left(e_{h},\nabla \eta \right)}_{\Omega }+\tau_{K}\sum_{K\in T_{h}}{\left(\nabla \eta_{h},\nabla \eta \right)}_{K}\\ \leq & [\sum_{K\in T_{h}}\nu_{1}∥e_{h}{∥}_{1,K}^{2}+\;\nu_{2}∥\theta_{h}{∥}_{1,K}^{2}+\;4\nu_{r}∥\theta_{h}{∥}_{0,K}^{2}+\;2\nu_{r}∥\theta_{h}{∥}_{1,K}^{2}+\;2\nu_{r}{∥e_{h}∥}_{1,K}^{2}\\ \; & +\;∥\eta_{h}{∥}_{0,K}^{2}+\;\frac{1}{\tau_{K}}∥e_{h}{∥}_{0,K}^{2}+\;\tau_{K}{∥\eta_{h}∥}_{1,K}{]}^{\frac{1}{2}}\\ \; & \cdot \;[\sum_{K\in T_{h}}\nu_{1}{∥e∥}_{1,K}^{2}+\;\nu_{2}{∥\theta ∥}_{1,K}^{2}+\;4\nu_{r}{∥\theta ∥}_{0,K}^{2}+2\nu_{r}{∥e∥}_{0,K}^{2}+2\nu_{r}{∥\theta ∥}_{0,K}^{2}\\ \; & +\;{∥\mathrm{div}\;e∥}_{0,K}^{2}+\tau_{K}{∥\eta ∥}_{1,K}^{2}+\tau_{K}{∥\eta ∥}_{1,K}^{2}{]}^{\frac{1}{2}}\\ \leq & c[\sum_{K\in T_{h}}\left(\nu_{1}+2\nu_{r}\right)∥e_{h}{∥}_{1,K}^{2}+\;\frac{1}{\tau_{K}}∥e_{h}{∥}_{0,K}^{2}+\left(\nu_{2}+2\nu_{r}\right)∥\theta_{h}{∥}_{1,K}^{2}+\;4\nu_{r}{∥\theta_{h}∥}_{0,K}^{2}\\ \; & +\;∥\eta_{h}{∥}_{0,K}^{2}+\;\tau_{K}{∥\eta_{h}∥}_{1,K}^{2}{]}^{\frac{1}{2}}\\ \; & \cdot \;{\left[\sum_{K\in T_{h}}\left(\nu_{1}+1\right){∥e∥}_{1,K}^{2}+2\nu_{r}{∥e∥}_{0,K}^{2}+\nu_{2}{∥\theta ∥}_{1,K}^{2}+6\nu_{r}{∥\theta ∥}_{0,K}^{2}+\tau_{K}{∥\eta ∥}_{1,K}^{2}\right]}^{\frac{1}{2}}, \end{array}$$

from the above inequalities,

$$\begin{array}{cc} \; & \min\;\left\{\nu ,\nu_{2}\right\}\left({∥e∥}_{1}^{2}+{∥\theta ∥}_{1}^{2}+{∥\eta ∥}_{h}^{2}\right)\\ \leq & c[\sum_{K\in T_{h}}\left(\nu_{1}+2\nu_{r}\right)∥e_{h}{∥}_{1,K}^{2}+\;\frac{1}{\tau_{K}}∥e_{h}{∥}_{0,K}^{2}+\left(\nu_{2}+2\nu_{r}\right)∥\theta_{h}{∥}_{1,K}^{2}+\;4\nu_{r}{∥\theta_{h}∥}_{0,K}^{2}\\ \; & +\;∥\eta_{h}{∥}_{0,K}^{2}+\;\tau_{K}{∥\eta_{h}∥}_{1,K}^{2}{]}^{\frac{1}{2}}\\ \; & \cdot \;{\left[\sum_{K\in T_{h}}\left(\nu_{1}+1\right){∥e∥}_{1,K}^{2}+2\nu_{r}{∥e∥}_{0,K}^{2}+\nu_{2}{∥\theta ∥}_{1,K}^{2}+6\nu_{r}{∥\theta ∥}_{0,K}^{2}+\tau_{K}{∥\eta ∥}_{1,K}^{2}\right]}^{\frac{1}{2}}\\ \leq & c\sqrt{\max\;\{\nu_{1}+1,2\nu_{r},\nu_{2},6\nu_{r}\}}[\sum_{K\in T_{h}}\left(\nu_{1}+2\nu_{r}\right)∥e_{h}{∥}_{1,K}^{2}+\;\frac{1}{\tau_{K}}{∥e_{h}∥}_{0,K}^{2}\\ \; & +\left(\nu_{2}+2\nu_{r}\right)∥\theta_{h}{∥}_{1,K}^{2}+\;4\nu_{r}∥\theta_{h}{∥}_{0,K}^{2}+\;∥\eta_{h}{∥}_{0,K}^{2}+\;\tau_{K}{∥\eta_{h}∥}_{1,K}^{2}{]}^{\frac{1}{2}}\\ \; & \cdot \;{\left[{∥e∥}_{1,\Omega }^{2}+{∥\theta ∥}_{1,\Omega }^{2}+{∥\eta ∥}_{h,\Omega }^{2}\right]}^{\frac{1}{2}}, \end{array}$$

and hence, dividing by the last term we get

$$\begin{array}{cc} \; & {∥e∥}_{1}+{∥\theta ∥}_{1}+{∥\eta ∥}_{h}\\ \leq & c\frac{\sqrt{\max\;\{\nu_{1}+1,2\nu_{r},c_{1},6\nu_{r}\}}}{\min\;\{\nu ,\nu_{2}\}}[\sum_{K\in T_{h}}\left(\nu_{1}+2\nu_{r}\right)∥e_{h}{∥}_{1,K}^{2}+\;\frac{1}{\tau_{K}}{∥e_{h}∥}_{0,K}^{2}\\ \; & +\left(\nu_{2}+2\nu_{r}\right)∥\theta_{h}{∥}_{1,K}^{2}+\;4\nu_{r}∥\theta_{h}{∥}_{0,K}^{2}+\;∥\eta_{h}{∥}_{0,K}^{2}+\;\tau_{K}{∥\eta_{h}∥}_{1,K}^{2}{]}^{\frac{1}{2}}\\ \leq & ch(∥u{∥}_{2,\Omega }+∥\omega {∥}_{2,\Omega }+∥p{∥}_{1,\Omega }) \end{array}$$

Finally, since $u-u_{h}=e_{h}-e,\omega -\omega_{h}=\theta_{h}-\theta$ and $p-p_{h}=\eta_{h}-\eta$, with the triangle inequality yields

$$\begin{array}{c} ∥u\;-\;u_{h}{∥}_{1,\Omega }+∥\omega \;-\;\omega_{h}{∥}_{1,\Omega }+∥p\;-\;p_{h}{∥}_{h}\;\leq {ch(∥u∥}_{2,\Omega }+{∥\omega ∥}_{2,\Omega }+{∥p∥}_{1,\Omega }). \end{array}$$

□

#### An Improved Error Estimate

Noting that the above estimation of pressure is still depend on the mesh parameter *h*, we can modified it into mesh independent. The main idea of proof can refer to the similar result of [9,21].

**Theorem** **8.** *Let $\left(u,\omega ,p\right)\in {\left[H^{2}\left(\Omega \right)\cap H_{0}^{1}\left(\Omega \right)\right]}^{n}\times {\left[H^{2}\left(\Omega \right)\cap H_{0}^{1}\left(\Omega \right)\right]}^{n}\times \left[H^{1}\left(\Omega \right)\cap L_{0}^{2}\left(\Omega \right)\right]$ be the solution of *(1)*. Then, the error satisfies*

$$\begin{array}{c} ∥u\;-\;u_{Rh}{∥}_{1,\Omega }+∥\omega \;-\;\omega_{Rh}{∥}_{1,\Omega }+∥p\;-\;p_{Rh}{∥}_{0,\Omega }\;\leq {ch(∥u∥}_{2,\Omega }+{∥\omega ∥}_{2,\Omega }+{∥p∥}_{1,\Omega }). \end{array}$$

**Proof.**  Noting that, if we choose $\tau_{Z}=0$ in the multiscale enrichment method of the next subsection, then we can recover the regular method from the multiscale enrichment method. The proof is omitted. □

### 3.3. Multiscale Enrichment Method

Find $\left(u_{Mh},\omega_{Mh},p_{Mh}\right)\in \left(X_{h},W_{h},M_{h}\right)$, such that

(42) $$B_{3}\left(\left(u_{Mh},\omega_{Mh},p_{Mh}\right),\left(v,s,q\right)\right)=F\left(v,s,q\right),$$

where

(43) $$\begin{array}{cc} \; & B_{3}\left(\left(u_{Mh},\omega_{Mh},p_{Mh}\right),\left(v,s,q\right)\right)\\ = & a_{1}\left(u_{Mh},v\right)+a_{2}\left(\omega_{Mh},s\right)+4\nu_{r}\left(\omega_{Mh},s\right)-\left(\mathrm{div}\;v,p_{Mh}\right)+\left(\mathrm{div}\;u_{Mh},q\right)\\ \; & -\;2\nu_{r}\left(\mathrm{rot}\;\omega_{Mh},v\right)-2\nu_{r}\left(\mathrm{rot}\;u_{Mh},s\right)\\ \; & +\sum_{K\in T_{h}}\tau_{K}{\left(\nabla p_{Mh}-2\nu_{r}\mathrm{rot}\;\omega_{Mh},\nabla q\right)}_{K}\\ \; & +\sum_{Z\in \Gamma_{kj}}\tau_{Z}{\left(\left[\nu \partial_{n}u_{Mh}\right],\left[\nu \partial_{n}v\right]\right)}_{Z}, \end{array}$$

(44) $$F\left(v,s,q\right)={\left(f,v\right)}_{\Omega }+{\left(g,s\right)}_{\Omega }+\sum_{K\in T_{h}}\tau_{K}{\left(f,\nabla q\right)}_{K},$$

(45) $$\tau_{K}=\beta_{K}\frac{h_{K}^{2}}{\nu_{1}},\;\tau_{Z}=\beta_{e}\frac{h_{Z}}{\nu_{1}},$$

where $\tau_{K}$ and $\tau_{Z}$ are the positive stabilization parameters, the quantity $h_{Z}=\left|Z\right|$ is the length of the edge Z,Z⊂∂K, and [v] denotes the jump of *v* across $\Gamma_{kj}$. Define the mesh-dependent norms

(46) $$\begin{array}{cc} {\left|||v||\right|}_{h}^{2} & =\nu_{1}{∥v∥}_{1,\Omega }^{2}+\sum_{z\in \Gamma_{kj}}\tau_{Z}{∥\left[\nu \partial_{n}v\right]∥}_{0,Z}^{2}, \end{array}$$

(47) $$\begin{array}{cc} {∥q∥}_{h}^{2} & =\sum_{K\in T_{h}}\tau_{K}{∥q∥}_{1,K}^{2}. \end{array}$$

Before analyzing the stability of the method given by (42), we introduce the following local trace theorems

(48) $$\begin{array}{c} {∥v∥}_{0,\partial K}^{2}\leq c(h_{K}^{-1}{∥v∥}_{0,K}^{2}+h_{K}{∥v∥}_{1,K}^{2}). \end{array}$$

**Lemma** **3.** *The bilinear form $B_{3}\left(\left(u_{Mh},\omega_{Mh},p_{Mh}\right),\left(v,s,q\right)\right)$ satisfies the continuous property*

(49) $$\begin{array}{c} B_{3}\left(\left(u_{Mh},\omega_{Mh},p_{Mh}\right),\left(v,s,q\right)\right)\leq c(∥u_{Mh}{∥}_{1}+\;∥\omega_{Mh}{∥}_{+}∥p_{Mh}{∥}_{0}{)(∥v∥}_{1}+{∥s∥}_{1}+{∥q∥}_{0}). \end{array}$$

*for all $\left(u_{Mh},\omega_{Mh},p_{Mh}\right)\in \left(X_{h},X_{h},M_{h}\right)$, $\left(v,s,q\right)\in \left(X_{h},X_{h},M_{h}\right)$, and the elliptical property*

(50) $$\begin{array}{cc} \; & B_{3}\left(\left(u_{Mh},\omega_{Mh},p_{Mh}\right),\left(u_{Mh},\omega_{Mh},p_{Mh}\right)\right)\\ \geq & c(∥u_{Mh}{∥}_{1,\Omega }^{2}+\sum_{Z\in \Gamma_{kj}}\tau_{Z}∥\left[\nu_{1}\partial_{n}u_{Mh}\right]{∥}_{0,Z}^{2}+∥\omega_{Mh}{∥}_{1,\Omega }^{2}+∥p_{Mh}{∥}_{h}^{2}). \end{array}$$

**Proof.**  The continuous property follows using (48) and Cauchy-Schwartz inequality.

$$\begin{array}{cc} \; & B_{3}{\left(\left(u_{Mh},\omega_{Mh},p_{Mh}\right),\left(u_{Mh},\omega_{Mh},p_{Mh}\right)\right)}_{K}\\ = & \nu_{1}∥u_{Mh}{∥}_{1,K}^{2}+\;\nu_{2}∥\omega_{Mh}{∥}_{1,K}^{2}+4\;\nu_{r}∥\omega_{Mh}{∥}_{0,K}^{2}+\;\tau_{K}{∥p_{Mh}∥}_{0,K}^{2}\\ \; & -\;4\nu_{r}{\left(\omega_{Mh},\mathrm{rot}\;u_{Mh}\right)}_{K}+\sum_{Z\in \Gamma_{kj}}\tau_{Z}{∥\left[\nu_{1}\partial_{n}u_{Mh}\right]∥}_{0,Z}^{2}\\ \geq & \nu_{1}∥u_{Mh}{∥}_{1,K}^{2}+\;\nu_{2}∥\omega_{Mh}{∥}_{1,K}^{2}+\;4\nu_{r}∥\omega_{Mh}{∥}_{0,K}^{2}+\;\tau_{K}{∥p_{Mh}∥}_{0,K}^{2}\\ \; & -\;4\nu_{r}∥\omega_{Mh}{∥}_{0,K}∥\nabla u_{Mh}{∥}_{0,K}+\sum_{Z\in \Gamma_{kj}}\tau_{Z}{∥\left[\nu_{1}\partial_{n}u_{Mh}\right]∥}_{0,Z}^{2}. \end{array}$$

Since $2ab\leq \frac{1}{\epsilon }a^{2}+\epsilon b^{2}$ with ϵ>0, we see that

$$\begin{array}{cc} \; & B_{3}{\left(\left(u_{Mh},\omega_{Mh},p_{Mh}\right),\left(u_{Mh},\omega_{Mh},p_{Mh}\right)\right)}_{K}\\ \geq & \left(\nu_{1}-2\nu_{r}\epsilon \right)∥u_{Mh}{∥}_{1,K}^{2}+\;\nu_{2}∥\omega_{Mh}{∥}_{1,K}^{2}+\left(4\nu_{r}-\frac{2\nu_{r}}{\epsilon }\right){∥\omega_{Mh}∥}_{0,K}^{2}\\ \; & +\sum_{K\in T_{h}}\tau_{K}∥p_{Mh}{∥}_{1,K}^{2}+\sum_{Z\in \Gamma_{kj}}\tau_{Z}{∥\left[\nu_{1}\partial_{n}u_{Mh}\right]∥}_{0,Z}^{2}. \end{array}$$

Finally, taking $\epsilon =\frac{1}{2}$ to obtain

$$\begin{array}{cc} \; & B_{3}{\left(\left(u_{Mh},\omega_{Mh},p_{Mh}\right),\left(u_{Mh},\omega_{Mh},p_{Mh}\right)\right)}_{K}\\ \geq & \nu ∥u_{Mh}{∥}_{1,K}^{2}+\;\nu_{2}∥\omega_{Mh}{∥}_{1,K}^{2}+\sum_{K\in T_{h}}\tau_{K}∥p_{Mh}{∥}_{1,K}^{2}+\sum_{Z\in \Gamma_{kj}}\tau_{Z}{∥\left[\nu_{1}\partial_{n}u_{Mh}\right]∥}_{0,Z}^{2}\\ \geq & c(∥u_{Mh}{∥}_{1,K}^{2}+\sum_{Z\in \Gamma_{kj}}\tau_{Z}∥\left[\nu_{1}\partial_{n}u_{Mh}\right]{∥}_{0,Z}^{2}+∥\omega_{Mh}{∥}_{1,K}^{2}+∥p_{Mh}{∥}_{h}^{2}). \end{array}$$

□

Next, we introduce the interpolation error about the Clément interpolation operator.

**Lemma** **4.** *Let $\left(v,s,q\right)\in {\left[H^{2}\left(\Omega \right)\cap H_{0}^{1}\left(\Omega \right)\right]}^{2}\times {\left[H^{2}\left(\Omega \right)\cap H_{0}^{1}\left(\Omega \right)\right]}^{2}\times \left[H^{1}\left(\Omega \right)\cap L_{0}^{2}\left(\Omega \right)\right]$, and ${\tilde{q}}_{h}=C_{h}\left(q\right)-\frac{{\left(C_{h}\left(q\right),1\right)}_{\Omega }}{\left|\Omega \right|}$, such that*

(51) $$\begin{array}{c} |||v-I_{h}{v|||}_{h}^{2}\;+\sum_{K\in T_{h}}\tau_{K}^{-1}∥v\;-\;I_{h}{v∥}_{0,K}^{2}\leq ch^{2}\nu_{1}{∥v∥}_{2,\Omega }^{2}, \end{array}$$

(52) $$\begin{array}{c} ∥q\;-\;{\tilde{q}}_{h}{∥}_{h}+\;\frac{1}{\sqrt{\nu_{1}}}∥q\;-\;{\tilde{q}}_{h}{∥}_{0,\Omega }\;\leq ch\frac{1}{\sqrt{\nu_{1}}}{∥q∥}_{1,\Omega }. \end{array}$$

**Proof.**  The result comes from the norm definition and uses $∥q\;-\;{\tilde{q}}_{h}{∥}_{0,\Omega }\;\leq {∥q-C_{h}\left(q\right)∥}_{0,\Omega }$ to combine with (15) and (16). □

**Theorem** **9.** *Let $\left(u,\omega ,p\right)\in {\left[H^{2}\left(\Omega \right)\cap H_{0}^{1}\left(\Omega \right)\right]}^{n}\times {\left[H^{2}\left(\Omega \right)\cap H_{0}^{1}\left(\Omega \right)\right]}^{n}\times \left[H^{1}\left(\Omega \right)\cap L_{0}^{2}\left(\Omega \right)\right]$ be the solution of *(1)*, and $\left(u_{Mh},\omega_{Mh},p_{Mh}\right)$ the solution of *(42)*. Then, the following error estimate holds*

$$\begin{array}{cc} \; & |||u-u_{Mh}{\left||\right|}_{h}\;+∥\omega \;-\;\omega_{Mh}{∥}_{1,\Omega }+∥p\;-\;p_{Mh}{∥}_{h}\;\leq ch(\sqrt{\nu_{1}}{∥u∥}_{2,\Omega }+{∥\omega ∥}_{2,\Omega }+\frac{1}{\sqrt{\nu_{1}}}{∥p∥}_{1,\Omega }). \end{array}$$

**Proof.**  For the sake of simplicity, let ${\tilde{u}}_{h}=I_{h}u,{\tilde{\omega }}_{h}=I_{h}\omega$, $\left(\eta_{h}^{u},\eta_{h}^{\omega },\eta_{h}^{p}\right)=\left(u-{\tilde{u}}_{h},\omega -{\tilde{\omega }}_{h},p-{\tilde{p}}_{h}\right)$. Then

$$\begin{array}{cc} \; & \nu_{1}∥u_{Mh}-{\tilde{u}}_{h}{∥}_{1}^{2}+\sum_{Z\in \Gamma_{kj}}\tau_{Z}∥\nu_{1}\partial_{n}\left(u_{Mh}-{\tilde{u}}_{h}\right){∥}_{0,Z}^{2}+\;\nu_{2}∥\omega_{Mh}-{\tilde{\omega }}_{h}{∥}_{1}^{2}+\;{∥p_{Mh}-{\tilde{p}}_{h}∥}_{h}^{2}\\ \leq & B_{3}\left(\left(u_{Mh}-{\tilde{u}}_{h},\omega_{Mh}-{\tilde{\omega }}_{h},p_{Mh}-{\tilde{p}}_{h}\right),\left(u_{Mh}-{\tilde{u}}_{h},\omega_{Mh}-{\tilde{\omega }}_{h},p_{Mh}-{\tilde{p}}_{h}\right)\right)\\ = & B_{3}\left(\left(\eta_{h}^{u},\eta_{h}^{\omega },\eta_{h}^{p}\right),\left(u_{Mh}-{\tilde{u}}_{h},\omega_{Mh}-{\tilde{\omega }}_{h},p_{Mh}-{\tilde{p}}_{h}\right)\right)\\ = & \nu_{1}{\left(\nabla \eta_{h}^{u},\nabla \left(u_{Mh}-{\tilde{u}}_{h}\right)\right)}_{\Omega }+\nu_{2}{\left(\nabla \eta_{h}^{\omega },\nabla \left(\omega_{Mh}-{\tilde{\omega }}_{h}\right)\right)}_{\Omega }+4\nu_{r}{\left(\eta_{h}^{\omega },\omega_{Mh}-{\tilde{\omega }}_{h}\right)}_{\Omega }\\ \; & -\;{\left(\mathrm{div}\;\left(u_{Mh}-{\tilde{u}}_{h}\right),\eta_{h}^{p}\right)}_{\Omega }-{\left(\eta_{h}^{u},\nabla \left(p_{Mh}-{\tilde{p}}_{h}\right)\right)}_{\Omega }-2\nu_{r}{\left(\mathrm{rot}\;\eta_{h}^{\omega },u_{Mh}-{\tilde{u}}_{h}\right)}_{\Omega }\\ \; & -\;2\nu_{r}{\left(\mathrm{rot}\;\eta_{h}^{u},\omega_{Mh}-{\tilde{\omega }}_{h}\right)}_{\Omega }+\sum_{K\in T_{h}}\tau_{K}{\left(\nabla \eta_{h}^{p},\nabla \left(p_{Mh}-{\tilde{p}}_{h}\right)\right)}_{K}\\ \; & +\sum_{Z\in \Gamma_{kj}}\tau_{Z}{\left(\left[\nu_{1}\partial_{n}\eta_{h}^{u}\right],\left[\nu_{1}\partial_{n}\left(u_{Mh}-{\tilde{u}}_{h}\right)\right]\right)}_{Z}\\ \leq & [\nu_{1}∥\eta_{h}^{u}{∥}_{1,\Omega }^{2}+\;\nu_{2}∥\eta_{h}^{\omega }{∥}_{1,\Omega }^{2}+\;4\nu_{r}∥\eta_{h}^{\omega }{∥}_{0,\Omega }^{2}+\;2\nu_{r}∥\eta_{h}^{\omega }{∥}_{0,\Omega }^{2}+\;2\nu_{r}∥\eta_{h}^{u}{∥}_{1,\Omega }^{2}+\;{∥\eta_{h}^{p}∥}_{0,\Omega }^{2}\\ \; & +\;∥\eta_{h}^{u}{∥}_{0,\Omega }^{2}+\;∥\eta_{h}^{p}{∥}_{h}^{2}+\sum_{Z\in \Gamma_{kj}}\tau_{Z}{∥\left[\nu_{1}\partial_{n}\eta_{h}^{u}\right]∥}_{0,Z}^{2}{]}^{\frac{1}{2}}\\ \; & \cdot \;[\nu_{1}∥u_{Mh}-{\tilde{u}}_{h}{∥}_{1,\Omega }^{2}+\;\nu_{2}∥\omega_{Mh}-{\tilde{\omega }}_{h}{∥}_{1,\Omega }^{2}+\;6\nu_{r}∥\omega_{Mh}-{\tilde{\omega }}_{h}{∥}_{0,\Omega }^{2}+\;2\nu_{1}{∥u_{Mh}-{\tilde{u}}_{h}∥}_{1,\Omega }^{2}\\ \; & +\;2\sum_{K\in T_{h}}\tau_{K}∥\nabla \left(p_{Mh}-{\tilde{p}}_{h}\right){∥}_{0,K}^{2}+\sum_{Z\in \Gamma_{kj}}\tau_{Z}{∥\left[\nu_{1}\partial_{n}\left(u_{Mh}-{\tilde{u}}_{h}\right)\right]∥}_{0,Z}^{2}{]}^{\frac{1}{2}}\\ \leq & c{\left[|∥\eta_{h}^{u}{∥|}_{h}^{2}+\nu_{2}∥\eta_{h}^{\omega }{∥}_{1,\Omega }^{2}+\;∥\eta_{h}^{u}{∥}_{0,\Omega }^{2}+\;6\nu_{r}∥\eta_{h}^{\omega }{∥}_{0,\Omega }^{2}+\;∥\eta_{h}^{p}{∥}_{0,\Omega }^{2}+\;{∥\eta_{h}^{p}∥}_{h}^{2}\right]}^{\frac{1}{2}}\\ \; & \cdot \;{\left[\left||\right|u_{Mh}-{\tilde{u}}_{h}{\left||\right|}_{h}^{2}+\nu_{2}∥\omega_{Mh}-{\tilde{\omega }}_{h}{∥}_{1,\Omega }^{2}+\;{∥p_{Mh}-{\tilde{p}}_{h}∥}_{h}^{2}\right]}^{\frac{1}{2}}. \end{array}$$

Hence, dividing by the last term we get

$$\begin{array}{cc} \; & \left||\right|u_{Mh}-{\tilde{u}}_{h}{\left||\right|}_{h}+\nu_{2}∥\omega_{Mh}-{\tilde{\omega }}_{h}{∥}_{1,\Omega }+\;{∥p_{Mh}-{\tilde{p}}_{h}∥}_{h}\\ \leq & c{\left[\left||\right|\eta_{h}^{u}{\left||\right|}_{h}^{2}+\nu_{2}∥\eta_{h}^{\omega }{∥}_{1,\Omega }^{2}+\;∥\eta_{h}^{u}{∥}_{0,\Omega }^{2}+\;6\nu_{r}∥\eta_{h}^{\omega }{∥}_{0,\Omega }^{2}+\;∥\eta_{h}^{p}{∥}_{0,\Omega }^{2}+\;{∥\eta_{h}^{p}∥}_{h}^{2}\right]}^{\frac{1}{2}}\\ \leq & ch(\sqrt{\nu_{1}}∥u{∥}_{2,\Omega }+∥\omega {∥}_{2,\Omega }+\;\frac{1}{\sqrt{\nu_{1}}}∥p{∥}_{1,\Omega }). \end{array}$$

Finally, combing above inequalities with the triangular inequality gives the following result.

$$\begin{array}{c} |||u-u_{Mh}{\left||\right|}_{h}+\nu_{2}∥\omega \;-\;\omega_{Mh}{∥}_{1,\Omega }+∥p\;-\;p_{Mh}{∥}_{h}\;\leq ch(\sqrt{\nu_{1}}{∥u∥}_{2,\Omega }+{∥\omega ∥}_{2,\Omega }+\frac{1}{\sqrt{\nu_{1}}}{∥p∥}_{1,\Omega }). \end{array}$$

□

**Remark** **1.** *Because of the norm definition, we cannot guarantee convergence of pressure. The next result shows that we have an independent optimal error estimate of h in the natural norm of pressure.*

**Theorem** **10.** *Let $\left(u,\omega ,p\right)\in {\left[H^{2}\left(\Omega \right)\cap H_{0}^{1}\left(\Omega \right)\right]}^{n}\times {\left[H^{2}\left(\Omega \right)\cap H_{0}^{1}\left(\Omega \right)\right]}^{2}\times \left[H^{1}\left(\Omega \right)\cap L_{0}^{n}\left(\Omega \right)\right]$ be the solution of *(1)*, and $\left(u_{Mh},\omega_{Mh},p_{Mh}\right)$ the solution of *(42)*. Then, the following error estimate holds*

$$\begin{array}{c} ∥p\;-\;p_{Mh}{∥}_{0,\Omega }\;\leq ch(\nu_{1}{∥u∥}_{2,\Omega }+{∥\omega ∥}_{2,\Omega }+{∥p∥}_{1,\Omega }). \end{array}$$

**Proof.**  Known by the continuous inf-sup condition, there exists φ∈X such that $\nabla \cdot \varphi =p-p_{Mh}$ and ${∥\varphi ∥}_{1,\Omega }\leq c{∥p-p_{Mh}∥}_{0,\Omega }$. Let $\varphi_{h}=C_{h}\left(\varphi \right)\in X_{h}$, we obtain

$$\begin{array}{cc} ∥p\;-\;p_{Mh}{∥}_{0,\Omega }^{2}\;= & {\left(\nabla \cdot \varphi ,p-p_{Mh}\right)}_{\Omega }={\left(\nabla \cdot \left(\varphi -\varphi_{h}\right),p-p_{Mh}\right)}_{\Omega }+{\left(\nabla \cdot \varphi_{h},p-p_{Mh}\right)}_{\Omega }\\ \leq & -\sum_{K\in T_{h}}{\left(\varphi -\varphi_{h},\nabla \left(p-p_{Mh}\right)\right)}_{K}+\nu_{1}{\left(\nabla \left(u-u_{Mh}\right),\nabla \varphi_{h}\right)}_{\Omega }\\ \; & -\;2\nu_{r}{\left(\mathrm{rot}\;\left(\omega -\omega_{Mh}\right),\varphi_{h}\right)}_{\Omega }+\sum_{Z\in \Gamma_{kj}}\tau_{Z}{\left(\left[\nu_{1}\partial_{n}\left(u-u_{Mh}\right)\right],\left[\nu_{1}\partial_{n}\varphi_{h}\right]\right)}_{0,Z}\\ \leq & \sum_{K\in T_{h}}∥\varphi \;-\;\varphi_{h}{∥}_{0,K}∥p\;-\;p_{Mh}{)∥}_{1,K}+\nu_{1}∥u\;-\;u_{Mh}{∥}_{1,\Omega }{∥\varphi_{Mh}∥}_{1,\Omega }\\ \; & +\;2\nu_{r}∥\omega \;-\;\omega_{Mh}{∥}_{1,\Omega }∥\varphi_{Mh}{∥}_{0,\Omega }+\sum_{Z\in \Gamma_{kj}}\tau_{Z}∥\left[\nu_{1}\partial_{n}\left(u-u_{Mh}\right)\right]{∥}_{0,Z}{∥\left[\nu_{1}\partial_{n}\varphi_{h}\right]∥}_{0,Z}\\ \leq & {\left[\sum_{K\in T_{h}}\tau_{K}^{-1}∥\varphi \;-\;\varphi_{h}{∥}_{0,K}^{2}+\;\nu_{1}∥\varphi_{h}{∥}_{1,\Omega }^{2}+\;2\nu_{r}∥\varphi_{h}{∥}_{1,\Omega }^{2}+\sum_{Z\in \Gamma_{kj}}\tau_{Z}{∥\left[\nu_{1}\partial_{n}\varphi_{h}\right]∥}_{0,Z}^{2}\right]}^{\frac{1}{2}}\\ \; & \cdot \;[\sum_{K\in T_{h}}\tau_{K}∥p\;-\;p_{Mh}{)∥}_{1,K}^{2}+\nu_{1}∥u\;-\;u_{Mh}{∥}_{1,\Omega }^{2}+\;2\nu_{r}{∥\omega -\omega_{Mh}∥}_{1,\Omega }^{2}\\ \; & +\sum_{Z\in \Gamma_{kj}}\tau_{Z}{∥\left[\nu_{1}\partial_{n}\left(u-u_{Mh}\right)\right]∥}_{0,Z}^{2}{]}^{\frac{1}{2}}\\ \leq & c\sqrt{\nu_{1}}(|||u-u_{Mh}{\left||\right|}_{h}\;+∥\omega \;-\;\omega_{Mh}{∥}_{1,\Omega }+∥p\;-\;p_{Mh}{)∥}_{h}{)(∥\varphi ∥}_{1,\Omega }^{2}\;+\;∥\varphi_{h}{{∥}_{1,\Omega }^{2})}^{\frac{1}{2}}\\ \leq & ch\sqrt{\nu_{1}}(\sqrt{\nu_{1}}{∥u∥}_{2,\Omega }+{∥\omega ∥}_{2,\Omega }+\frac{1}{\sqrt{\nu_{1}}}{∥p∥}_{1,\Omega })∥p-p_{Mh}{∥}_{0,\Omega }, \end{array}$$

Then divide by the last term to get the result. □

### 3.4. Local Gauss Integration Method

The center idea of this method is to add two local Gauss integrals to the original discrete formulation, seek $\left(u_{Gh},\omega_{Gh},p_{Gh}\right)\in \left(X_{h},X_{h},M_{h}\right)$ such that

(53) $$\left\{\begin{array}{c} a_{1}\left(u_{Gh},v\right)-d\left(v,p_{Gh}\right)+d\left(u_{Gh},q\right)+G\left(p_{Gh},q\right)=2\nu_{r}\left(\mathrm{rot}\;\omega_{Gh},v\right)+\left(f,v\right),\\ a_{2}\left(\omega_{Gh},s\right)+4\nu_{r}\left(\omega_{Gh},s\right)=2\nu_{r}\left(\mathrm{rot}\;u_{Gh},s\right)+\left(g,s\right), \end{array}\right.$$

(54) $$B_{4}\left(\left(u_{Gh},\omega_{Gh},p_{Gh}\right),\left(v,s,q\right)\right)=F\left(v,s\right),$$

where

(55) $$\begin{array}{cc} \; & B_{4}\left(\left(u_{Gh},\omega_{Gh},p_{Gh}\right),\left(v,s,q\right)\right)\\ = & B(\left(u_{Gh},\omega_{Gh},p_{Gh}\right),\left(\left(v,s,q\right)\right)-G\left(p_{Gh},q\right)\\ = & \nu_{1}\left(\nabla u_{Gh},\nabla v\right)+\nu_{2}\left(\nabla \omega_{Gh},\nabla s\right)-\left(\mathrm{div}\;v,p_{Gh}\right)+\left(\mathrm{div}\;u_{Gh},q\right)\\ \; & +\;4\nu_{r}\left(\omega_{Gh},s\right)-2\nu_{r}\left(\mathrm{rot}\;\omega_{Gh},v\right)-2\nu_{r}\left(\mathrm{rot}\;u_{Gh},s\right)-G\left(p_{Gh},q\right), \end{array}$$

(56) F(v,s)=(f,v)+(g,s),

$G(p_{Gh},q)$ is defined by

(57) $$G\left(p_{Gh},q\right)=\left(p_{Gh}-\Pi p_{Gh},q-\Pi q\right),$$

for all $\left(v,s,q\right)\in \left(X_{h},X_{h},M_{h}\right)$, and Π is a $L^{2}$ projection operator with the following properties:

(58) $$\left(p,q\right)=\left(\Pi p,q\right),\;\forall p\in L^{2}\left(\Omega \right),q\in R_{0},$$

(59) $${∥\Pi p∥}_{0}\leq c{∥p∥}_{0},\;\forall p\in L^{2}\left(\Omega \right),$$

(60) $${∥p-\Pi p∥}_{0}\leq ch{∥p∥}_{1},\;\forall p\in H^{1}\left(\Omega \right),$$

where $R_{0}\subset L^{2}\left(\Omega \right)$ denotes the piecewise constant space associated with the triangulation $T_{h}$.

Obviously, this method does not require a complex computation or a stabilization parameter. It is only necessary to compute the block $G_{e}$ with simple Gauss integrals. The stabilization term is defined as follows:

(61) $$G\left(p_{Gh},q\right)=\sum_{K\in K_{h}}\left\{\int_{K,2}p_{Gh}q\mathrm{dx}-\int_{K,1}p_{Gh}q\mathrm{dx}\right\},$$

where $\int_{K,i}p_{Gh}qdx$ indicates a local Gauss integral over *K* that is exact for polynomials of degree i,i=1,2. Obviously, the bilinear form $G(p_{Gh},q)$ is symmetric and semi-definitely generated on each local set *K*.

**Theorem** **11.** *The bilinear form $B_{4}\left(\left(u_{Gh},\omega_{Gh},p_{Gh}\right),\left(v,s,q\right)\right)$ satisfies the continuous property*

(62) $$\begin{array}{c} B_{4}\left(\left(u_{Gh},\omega_{Gh},p_{Gh}\right),\left(v,s,q\right)\right)\leq c(∥u_{Gh}{∥}_{1}+\;∥\omega_{Gh}{∥}_{1}+\;∥p_{Gh}{∥}_{0}{)(∥v∥}_{1}+{∥s∥}_{1}+{∥q∥}_{0}) \end{array}$$

*where $\forall \left(u_{Gh},\omega_{Gh},p_{Gh}\right),\left(v,s,q\right)\in \left(X_{h},X_{h},M_{h}\right)$, and the coercive property*

(63) $$\begin{array}{cc} \underset{\left(v,s,q\right)\in X_{h},W_{h},M_{h}}{\sup}\frac{|B_{4}\left(\left(u_{Gh},\omega_{Gh},p_{Gh}\right),\left(v,s,q\right)\right)|}{{(∥v∥}_{1}^{2}+{∥s∥}_{1}^{2}+{∥q∥}_{0}^{2}{)}^{\frac{1}{2}}}\geq \; & \tilde{\beta }(∥u_{Gh}{∥}_{1}^{2}+\;∥\omega_{Gh}{∥}_{1}^{2}+\;∥p_{Gh}{{∥}_{0}^{2})}^{\frac{1}{2}},\\ \left(u_{Gh},\omega_{Gh},p_{Gh}\right)\in \left(X_{h},X_{h},M_{h}\right), \end{array}$$

*where $\tilde{\beta }$ is a positive constant depending only on* Ω.

**Proof.**  $$\begin{array}{cc} \; & |B_{4}\left(\left(u_{Gh},\omega_{Gh},p_{Gh}\right),\left(v,s,q\right)\right)|\\ = & |\nu_{1}\left(\nabla u_{Gh},\nabla v\right)+\nu_{2}\left(\nabla \omega_{Gh},\nabla s\right)-d\left(v,p_{Gh}\right)+d\left(u_{Gh},q\right)\\ \; & +\;4\nu_{r}\left(\omega_{Gh},s\right)-2\nu_{r}\left(\mathrm{rot}\;\omega_{Gh},v\right)-2\nu_{r}\left(\mathrm{rot}\;u_{Gh},s\right)-G\left(p_{Gh},q\right)|\\ \leq & c(∥u_{Gh}{∥}_{1}∥v{∥}_{1}+∥\omega_{Gh}{∥}_{1}∥s{∥}_{1}+∥u_{Gh}{∥}_{1}∥s{∥}_{1}+∥\omega_{Gh}{∥}_{1}∥v{∥}_{1}\\ \; & +\;{∥v∥}_{1}∥p_{Gh}{∥}_{0}+\;∥u_{Gh}{∥}_{1}{∥q∥}_{0}\;+\;∥p_{Gh}{∥}_{0}{∥q∥}_{0})\\ \leq & c(∥u_{Gh}{∥}_{1}+\;∥\omega_{Gh}{∥}_{1}+\;∥p_{Gh}{∥}_{0}{)(∥v∥}_{1}+{∥s∥}_{1}+{∥q∥}_{0}). \end{array}$$

Thus it suffices to show the continuous property.

For the coercive property of $B_{4}$, $\forall p_{Gh}\in M_{h}$, there exists a positive constant $C_{0}$ and z∈X such that [2]

(64) $$\begin{array}{cc} \left(\mathrm{div}\;z,p_{Gh}\right) & =\;∥p_{Gh}{∥}_{0}^{2}, \end{array}$$

(65) $$\begin{array}{cc} {∥z∥}_{1} & \leq c_{0}{∥p_{Gh}∥}_{0}. \end{array}$$

Setting the finite element approximation $z_{Gh}\in X_{h}$ of *z*, we have

(66) $$\begin{array}{cc} ∥z_{Gh}{∥}_{1} & \leq c_{1}{∥p_{Gh}∥}_{0}. \end{array}$$

Then, for any $p_{Gh}\in M_{h}$, we choose any $\left(v,s,q\right)=\left(u_{Gh}-\lambda z_{Gh},\omega_{Gh},-p_{Gh}\right)$, where $0<\lambda <\frac{2(1-c_{1})}{c_{1}\nu_{r}}$.

Obviously, it follows from (58)–(61), (65) and (66) and the Young inequality that

(67) $$\begin{array}{cc} \; & B_{4}\left(\left(u_{Gh},\omega_{Gh},p_{Gh}\right),\left(v,s,q\right)\right)=B_{4}\left(\left(u_{Gh},\omega_{Gh},p_{Gh}\right),\left(u_{Gh}-\lambda z_{Gh},\omega_{Gh},p_{Gh}\right)\right)\\ = & a_{1}\left(u_{Gh},u_{Gh}\right)-\alpha a_{1}\left(u_{Gh},z_{Gh}\right)+a_{2}\left(\omega_{Gh},\omega_{Gh}\right)+\lambda d\left(z_{Gh},p_{Gh}\right)\\ \; & +\;4\nu_{r}\left(\omega_{Gh},\omega_{Gh}\right)-4\nu_{r}\left(\omega_{Gh},\mathrm{rot}u_{Gh}\right)+2\alpha \nu_{r}\left(\mathrm{rot}\omega_{Gh},z_{Gh}\right)+G\left(p_{Gh},p_{Gh}\right)\\ \geq & \nu_{1}∥u_{Gh}{∥}_{1}^{2}+\;\nu_{2}∥\omega_{Gh}{∥}_{1}^{2}+\;G\left(p_{Gh},p_{Gh}\right)+4\nu_{r}∥\omega_{Gh}{∥}_{0}^{2}-\;\lambda \nu_{1}∥u_{Gh}{∥}_{1}{∥z_{Gh}∥}_{1}\\ \; & -\;4\nu_{r}∥\omega_{Gh}{∥}_{0}∥u_{Gh}{∥}_{1}-\;2\nu_{r}∥\omega_{Gh}{∥}_{0}{∥z_{Gh}∥}_{1}+\lambda d\left(z_{Gh}-z,p_{Gh}\right)+\lambda d\left(z,p_{Gh}\right)\\ \geq & \frac{\nu }{2}∥u_{Gh}{∥}_{1}^{2}+\;\nu_{2}∥\omega_{Gh}{∥}_{1}^{2}+\;G\left(p_{Gh},p_{Gh}\right)+\lambda ∥p_{Gh}{∥}_{0}^{2}-\;\frac{\nu_{r}\alpha^{2}}{2}{∥z_{Gh}∥}_{1}^{2}\\ \; & -\;c_{2}\lambda ∥p_{Gh}-\Pi p_{Gh}{∥}_{1}{∥p_{Gh}∥}_{0}\\ \geq & \frac{\nu }{2}∥u_{Gh}{∥}_{1}^{2}+\;\nu_{2}∥\omega_{Gh}{∥}_{1}^{2}+\left(\lambda -\frac{c_{3}\nu_{r}\lambda^{2}}{2}-\frac{c_{4}\lambda }{2}\right){∥p_{Gh}∥}_{0}^{2}+\frac{1}{2}G\left(p_{Gh},p_{Gh}\right)\\ \geq & c_{5}(∥u_{Gh}{∥}_{1}^{2}+\;∥\omega_{Gh}{∥}_{1}^{2}+\;∥p_{Gh}{∥}_{0}^{2}). \end{array}$$

Finally, we remark that

$$\begin{array}{cc} {∥v∥}_{1}+{∥s∥}_{1}+{∥q∥}_{0} & =\;∥u_{Gh}-\lambda z_{Gh}{∥}_{1}+\;∥\omega_{Gh}{∥}_{1}+{∥p_{Gh}∥}_{0}\\ \; & \leq c_{6}(∥u_{Gh}{∥}_{1}^{2}+\;∥\omega_{Gh}{∥}_{1}^{2}+\;∥p_{Gh}{{∥}_{0}^{2})}^{\frac{1}{2}}, \end{array}$$

setting $\tilde{\beta }=\frac{c_{5}}{c_{6}}$, which finishes the proof. □

**Theorem** **12.** *Let $\left(u,\omega ,p\right)\in {\left[H^{2}\left(\Omega \right)\cap H_{0}^{1}\left(\Omega \right)\right]}^{n}\times {\left[H^{2}\left(\Omega \right)\cap H_{0}^{1}\left(\Omega \right)\right]}^{n}\times \left[H^{1}\left(\Omega \right)\cap L_{0}^{2}\left(\Omega \right)\right]$ be the solution of *(1)*, the stabilized finite element solution $\left(u_{Gh},\omega_{Gh},p_{Gh}\right)$ satisfies the error estimates:*

(68) $$∥u\;-\;u_{Gh}{∥}_{1}+∥\omega \;-\;\omega_{Gh}{∥}_{1}+∥p\;-\;p_{Gh}{∥}_{0}\;\leq {ch(∥u∥}_{2}+{∥\omega ∥}_{2}+{∥p∥}_{1}).$$

**Proof.**  Subtracting (54) from (6), we have

(69) $$\begin{array}{c} B_{4}\left(\left(u-u_{Gh},\omega -\omega_{Gh},p-p_{Gh}\right),\left(v,s,q\right)\right)=G\left(p,q\right). \end{array}$$

By setting $\left(e,\theta ,\eta \right)=\left(I_{h}u-u_{Gh},I_{h}\omega -\omega_{Gh},r_{h}p-p_{Gh}\right)$, it follows from, we can see that

(70) $$\begin{array}{cc} \; & B_{4}\left(\left(e,\theta ,\eta \right),\left(v,s,q\right)\right)\\ = & G\left(p,q\right)-B_{4}\left(\left(u-I_{h}u,\omega -I_{h}\omega ,p-r_{h}p\right),\left(v,s,q\right)\right)\\ \leq & \left|G\left(p,q\right)|\;+\;\right|B_{4}\left(\left(u-I_{h}u,\omega -I_{h}\omega ,p-r_{h}p\right),\left(v,s,q\right)\right)|\\ \leq & {c∥p-\Pi p∥}_{0}{∥q∥}_{0}+c(∥u-I_{h}{u∥}_{1}\;+∥\omega \;-\;I_{h}{\omega ∥}_{1}+∥p\;-\;r_{h}{p∥}_{0}{)(∥v∥}_{1}+{∥s∥}_{1}+{∥q∥}_{0})\\ \leq & {ch∥p∥}_{1}{(∥v∥}_{1}+{∥s∥}_{1}+{∥q∥}_{0}{)\;+\;ch(∥u∥}_{2}+{∥\omega ∥}_{2}+{∥p∥}_{1}{)(∥v∥}_{1}+{∥s∥}_{1}+{∥q∥}_{0})\\ \leq & {ch(∥u∥}_{2}+{∥\omega ∥}_{2}+{∥p∥}_{1}{)(∥v∥}_{1}+{∥s∥}_{1}+{∥q∥}_{0}). \end{array}$$

Obviously, it follows from (63) and (70) that

(71) $$\begin{array}{cc} \tilde{\beta }{(∥e∥}_{1}^{2}+{∥\theta ∥}_{1}^{2}+{∥\eta ∥}_{0}^{2}{)}^{\frac{1}{2}}\leq & \underset{0\neq \left(v,s,q\right)\in X_{h}\times W_{h}\times M_{h}}{\sup}\frac{|B_{4}\left(\left(e,\theta ,\eta \right)\right),\left(v,s,q\right))|}{{(∥v∥}_{1}^{2}+{∥s∥}_{1}^{2}+{∥q∥}_{0}^{2}{)}^{\frac{1}{2}}}\\ \leq & ch(∥u{∥}_{2}+∥\omega {∥}_{2}+∥p{∥}_{1}). \end{array}$$

Thanks to (71) and the triangle inequality

$$\begin{array}{cc} \; & ∥u\;-\;u_{Gh}{∥}_{1}+∥\omega \;-\;\omega_{Gh}{∥}_{1}+\;{∥p-p_{Gh}∥}_{1}\\ \leq & ∥u\;-\;I_{h}{u∥}_{1}\;+∥\omega \;-\;I_{h}{\omega ∥}_{1}\;+∥p\;-\;r_{h}{p∥}_{0}+{∥e∥}_{1}+{∥\theta ∥}_{1}+{∥\eta ∥}_{0}\\ \leq & ch(∥u{∥}_{2}+∥\omega {∥}_{2}+∥p{∥}_{1}). \end{array}$$

□

## 4. Numerical Experiments

**Figure 1:**
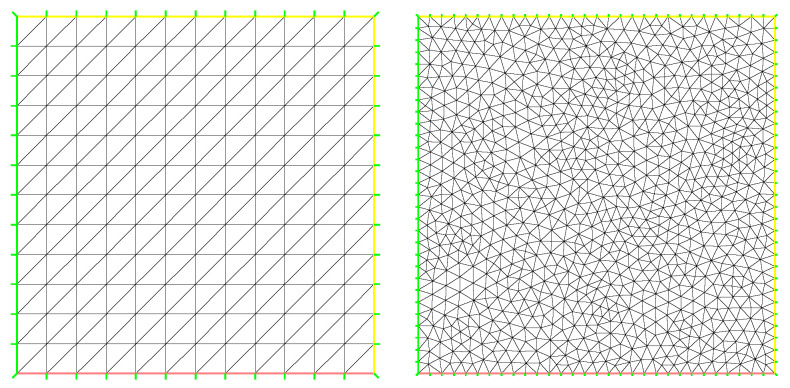
Two kinds of mesh.

In this section, we numerically compare the performance of the various stabilized mixed finite element methods discussed in the previous section for two examples. It is numerically solved by the stabilized mixed methods on uniform meshes (see the first picture in Figure 1). The second example solves the same problem on unstructured meshes (see the last picture in Figure 1). We recall that in our computations, the pressure and velocity are approximated by piecewise linear finite elements. All these computations have been based on the package FreeFem++, with some of our additional codes. The stabilized term of the regular must be controlled by carefully designed mesh-dependent parameters, whose optimal values are often unknown.

We now report the convergence rates for the penalty, regular, multiscale enrichment, and local Gauss integration methods for the steady micropolar equations solved by using the lowest equal-order element $p_{1}-p_{1}-p_{1}$ on the first uniform triangular mesh (see the first panel in Figure 1). The errors in the relative $L^{2}\left(\Omega \right)$- and $H^{1}\left(\Omega \right)$-norms for the velocity, angular velocity, and the relative $L^{2}\left(\Omega \right)$-norm for the pressure and their corresponding convergence rates are given in Table 1, Table 2, Table 3 and Table 4. In addition, the theoretical convergence rates should be of order $O(h^{2})$ and O(h) for the velocity and angular velocity in the $L^{2}\left(\Omega \right)$- and $H^{1}\left(\Omega \right)$-norms, respectively, and of order O(h) for the pressure in the $L^{2}\left(\Omega \right)$-norm by using all these stabilized methods. It follows from Table 1, Table 2, Table 3 and Table 4 that the theoretical results are confirmed for the velocity. Except for the penalty method, the speed and convergence order of the other methods have reached the results of theoretical analysis.

### 4.1. Problems with Smooth Solutions

Setting $\Omega ={\left[0,1\right]}^{2}$, equation parameters $\nu =\nu_{r}=c_{a}=c_{d}=0.1$, we take penalty parameter ε = $10^{-6}$ and $\varepsilon =O(h^{\frac{1}{2}})$ in Table 1 and Table 2 respectively, $\beta_{1}=100$, $\beta_{2}=100$, $\beta_{3}=150$, and the exact solution (u,ω,p) is with the right-hand side function *f* generated by the exact solution:

$$\begin{array}{cc} u_{1}\left(x,y\right)= & 10x^{2}{\left(x-1\right)}^{2}y\left(y-1\right)\left(2y-1\right),\\ u_{2}\left(x,y\right)= & -10x\left(x-1\right)\left(2x-1\right)y^{2}{\left(y-1\right)}^{2},\\ \omega (x,y)= & 10x^{2}{\left(x-1\right)}^{2}y\left(y-1\right)\left(2y-1\right)-10x\left(x-1\right)\left(2x-1\right)y^{2}{\left(y-1\right)}^{2},\\ p(x,y)= & 10(2x-1)(2y-1). \end{array}$$

It can be seen from Table 2, Table 3, Table 4 and Table 5 that, except for the multiscale enrichment method, the velocity and angular velocity convergence order of the other methods have reached the result of theoretical analysis. The velocity and angular velocity $L^{2}\left(\Omega \right)$-norm convergence order of the multiscale enrichment method do not reach $O(h^{2})$ with the refinement of the grid. The convergence speed of the $L^{2}\left(\Omega \right)$-norm of the pressure of the regular method, the local Gauss integration method, and the multiscale enrichment method has almost reached $O(h^{1.5})$, which are better than the expected results of the theoretical analysis.

Additionally, note the CPU time listed in Table 2, Table 3, Table 4 and Table 5, all our calculations are performed under the same computing platform. From Table 2, Table 3, Table 4 and Table 5, as the grid is encrypted, the multiscale enrichment method consumes the most time, while the local Gauss integration method consumes the least time.

In addition, take the other exact solution as follows:

$$\begin{array}{cc} u_{1}\left(x,y\right) & =\pi \sin^{2}\left(\pi x\right)\sin\left(2\pi y\right),\\ u_{2}\left(x,y\right) & =-\pi \sin\left(2\pi x\right)\sin^{2}\left(\pi y\right),\\ p(x,y) & =\cos(\pi x)\sin(\pi y),\\ \omega (x,y) & =\pi \sin^{2}\left(\pi x\right)\sin^{2}\left(\pi y\right). \end{array}$$

Given unstructured triangular mesh of Ω with different *h* = 0.270282, 0.14633, 0.073319, 0.0368164, 0.0188245, the errors are listed in Table 6, Table 7, Table 8 and Table 9.

### 4.2. The Lid-Driven Flow

**Figure 2:**
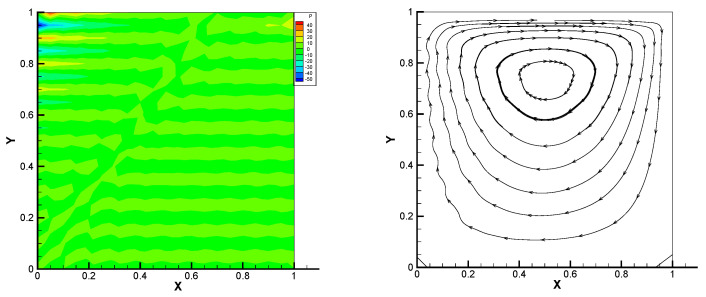
Pressure level lines and velocity streamlines for the penalty method.

**Figure 3:**
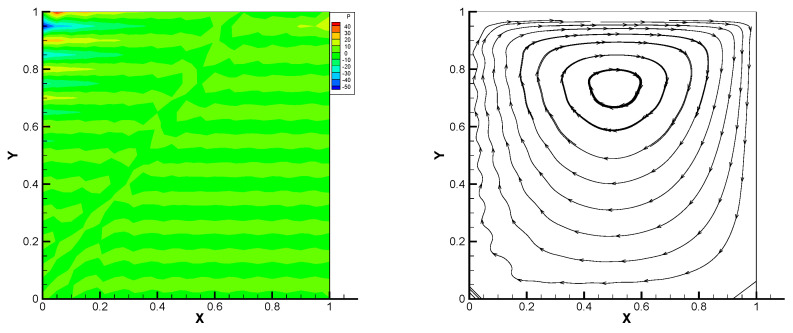
Pressure level lines and velocity streamlines for the regular method.

**Figure 4:**
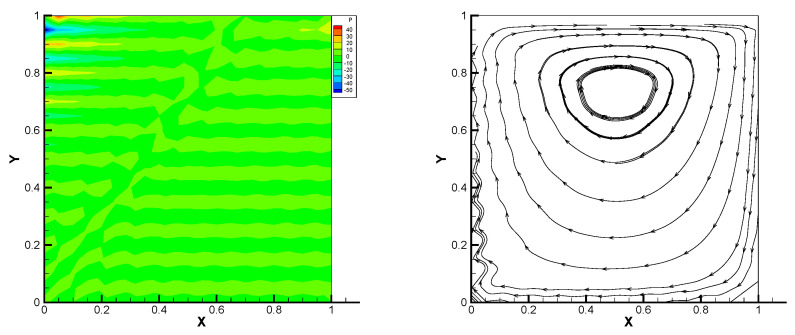
Pressure level lines and velocity streamlines for the multiscale enrichment method.

**Figure 5:**
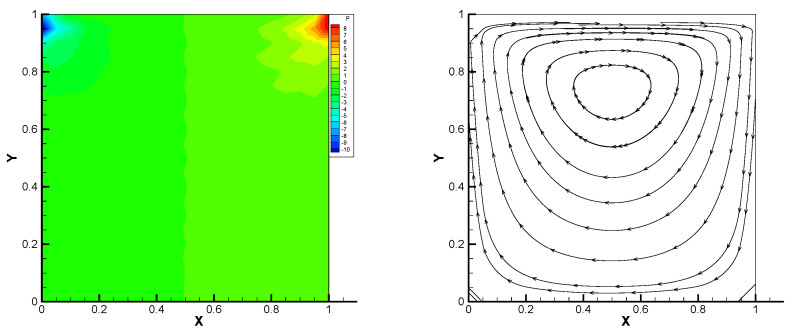
Pressure level lines and velocity streamlines for the local Gauss intergration method.

**Figure 6:**
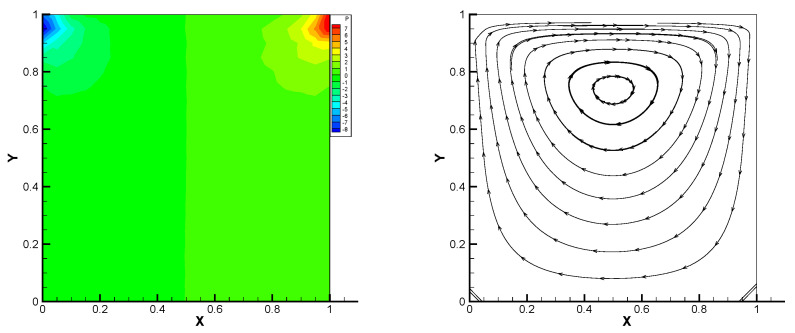
Pressure level lines and velocity streamlines for the local Gauss intergration method of p1b-p1-p1b.

This gives the lid-driven flow on a square area, which has a boundary condition of no-slip, and only at the upper boundary {(x,1):0<x<1} satisfies $u_{1}=1,u_{2}=0,w=1$. We assume that the normal component of velocity is zero on ∂Ω, and the tangential component is zero, except that y=1 is set to 1. In Figure 2, Figure 3, Figure 4, Figure 5 and Figure 6, we show the pressure levels and velocity streamlines with $\varepsilon =10^{-6},1/h=20$ based on these four methods. It can be seen from Figure 2, Figure 3, Figure 4, Figure 5 and Figure 6 that only the Gauss method can get the resolved pressure.

### 4.3. The Co-Axis Bearing Lubrication

**Figure 7:**
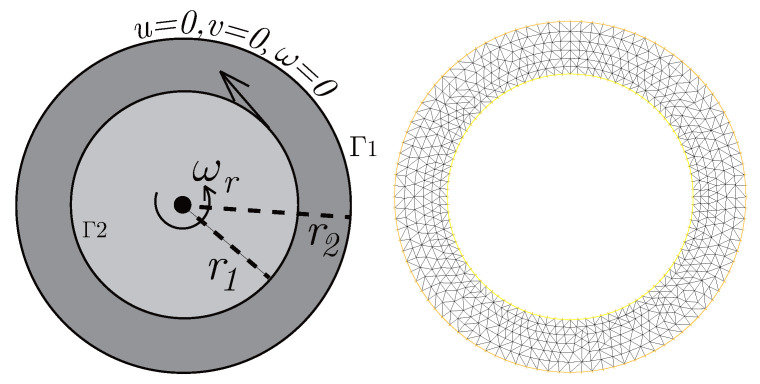
Survey region (**left**) and typical structured mesh (**right**).

In this example, we extend these stabilized methods to the nonlinear co-axis bearing lubrication problem. In Figure 7, the typical fluid domain, boundary conditions, and structured grid are drawn. The fluid domain is an angular domain between outer boundary $\Gamma_{1}$ with radius $r_{1}$ and inner boundary $\Gamma_{2}$ with radius $r_{2}$. The outer boundary is steady, and the inner body surrounded by $\Gamma_{2}$ is supposed to rotating along axis with rotating angular velocity $\omega_{r}$. So the homogeneous boundary condition $u_{x}=u_{y}=\omega =0$ on $\Gamma_{1}$, shearing velocity boundary condition $u_{x}=-r_{2}\omega_{r}\sin\left(\omega_{r}t\right),u_{y}=r_{2}\omega_{r}\cos\left(\omega_{r}t\right)$ and ω=0 on $\Gamma_{2}$ are added.

In this example, the parameters are selected as $r_{1}=1,r_{2}=0.5$, mesh size h=1/200, $j=\nu =\nu_{r}=c_{a}=c_{d}=0.1$, equation parameters $\nu =\nu_{r}=c_{a}=c_{d}=0.1$, $\varepsilon =10^{-8}$, $\beta_{1}=100$, $\beta_{2}=100$, $\beta_{3}=150$. Some numerical results with rotating angular velocity $\omega_{r}=100,500,1000$ are shown in Figure 8, Figure 9, Figure 10, Figure 11, Figure 12, Figure 13, Figure 14, Figure 15, Figure 16, Figure 17, Figure 18, Figure 19, Figure 20, Figure 21, Figure 22 and Figure 23. We observe that the pressure is well obtained by the local Gauss integration method.

From the above figures, as the rotation speed increases, the magnitude of fluid components horizontal velocity, vertical velocity, angular velocity, and pressure also increase. When $\omega_{r}=100$, the pressure of the local Gauss integration method performs better. Taking different values of $\omega_{r}$, the fluid field is still smooth, correspondingly, the non-linearity on the boundary also increases. It can also be applied to practical problems and has a wide range of applications.

## 5. Conclusions

In this paper, based on the lowest equal-order finite element space pair, a variety of stable mixed finite element methods are numerically studied for the stationary micropolar equations. The following conclusions are drawn through numerical comparison. The stability and efficiency of all these methods depend on their parameter values. As far as the penalty method is concerned, The smaller the parameter value, the more stable the method. However, for the regular and multiscale enrichment methods, their performance largely depends on the choice of the stabilization parameters. In fact, it is difficult to choose fine parameters. The local Gauss integration method has no stable parameters and shows the best performance among the considered methods on the numerical results.

## Figures and Tables

**Figure 8:**
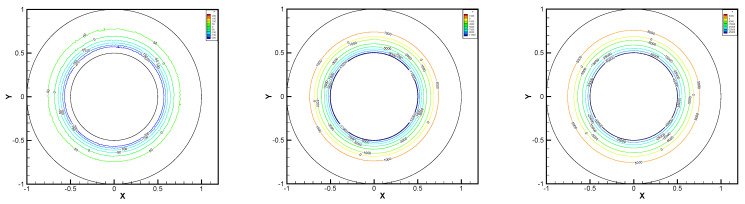
$\omega_{r}=100,500,1000$, pressure level lines for the penalty method.

**Figure 9:**
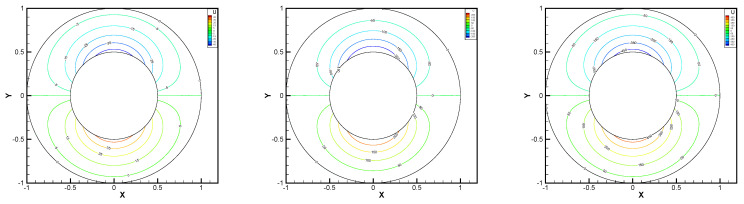
$\omega_{r}=100,500,1000$, horizontal velocity for the penalty method.

**Figure 10:**
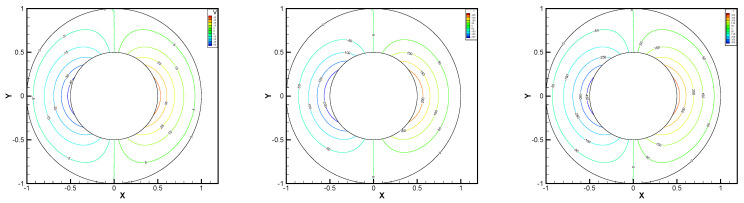
$\omega_{r}=100,500,1000$, vertical velocity for the penalty method.

**Figure 11:**
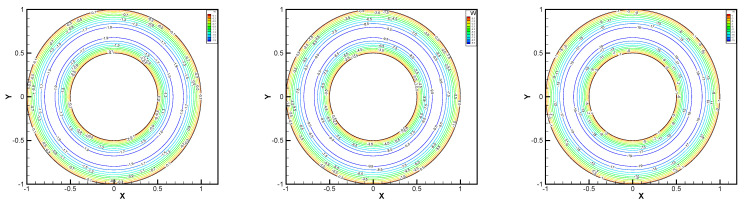
$\omega_{r}=100,500,1000$, angular velocity for the penalty method.

**Figure 12:**
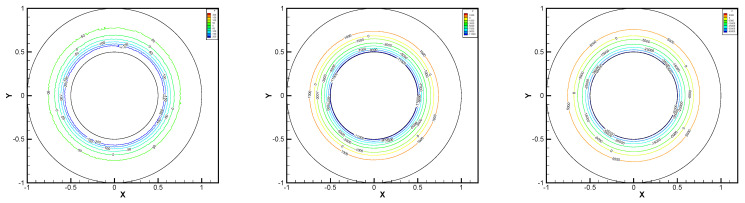
$\omega_{r}=100,500,1000$, pressure level lines for the regular method.

**Figure 13:**
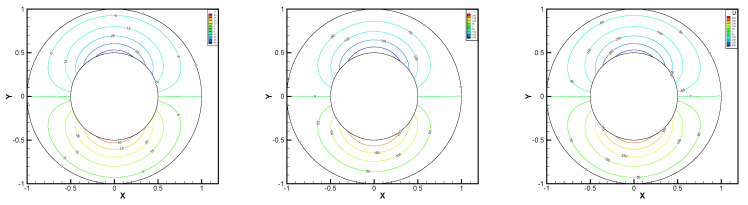
$\omega_{r}=100,500,1000$, horizontal velocity for the regular method.

**Figure 14:**
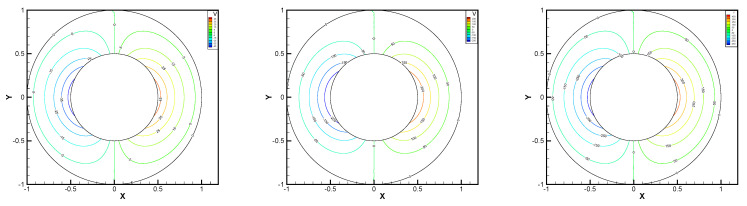
$\omega_{r}=100,500,1000$, vertical velocity for the regular method.

**Figure 15:**
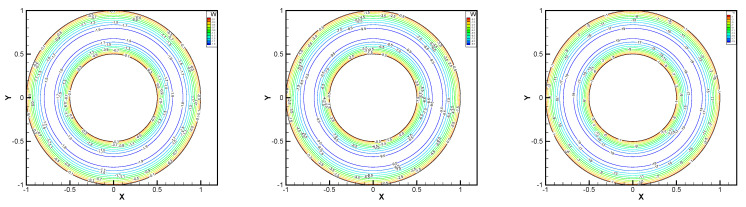
$\omega_{r}=100,500,1000$, angular velocity for the regular method.

**Figure 16:**
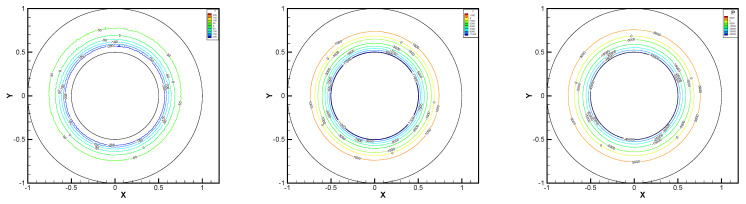
$\omega_{r}=100,500,1000$, pressure level lines for the multiscale enrichment method.

**Figure 17:**
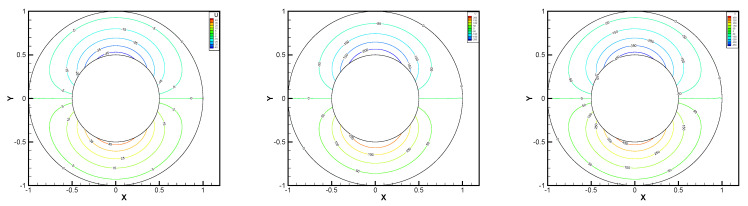
$\omega_{r}=100,500,1000$, horizontal velocity for the multiscale enrichment method.

**Figure 18:**
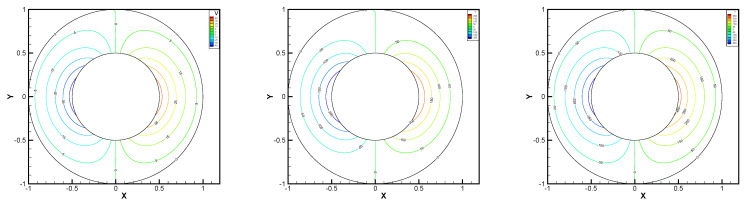
$\omega_{r}=100,500,1000$, vertical velocity for the multiscale enrichment method.

**Figure 19:**
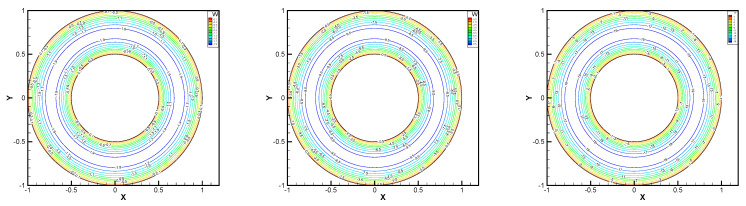
$\omega_{r}=100,500,1000$, angular velocity for the multiscale enrichment method.

**Figure 20:**
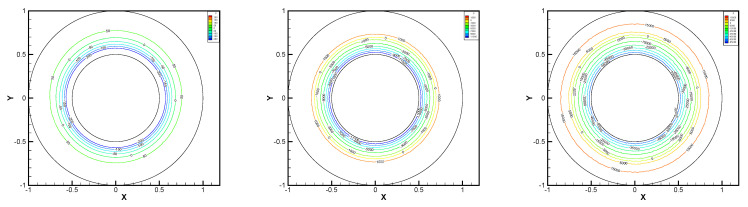
$\omega_{r}=100,500,1000$, pressure level lines for the local Gauss integration method.

**Figure 21:**
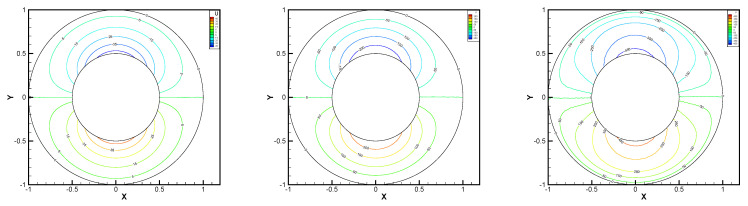
$\omega_{r}=100,500,1000$, horizontal velocity for the local Gauss integration method.

**Figure 22:**
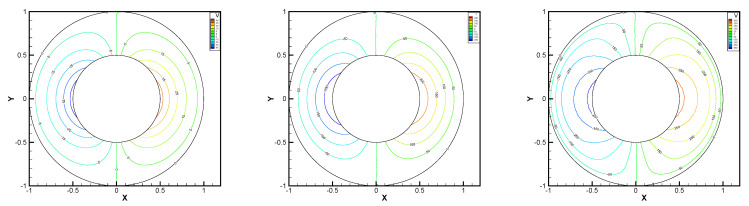
$\omega_{r}=100,500,1000$, vertical velocity for the local Gauss integration method.

**Figure 23:**
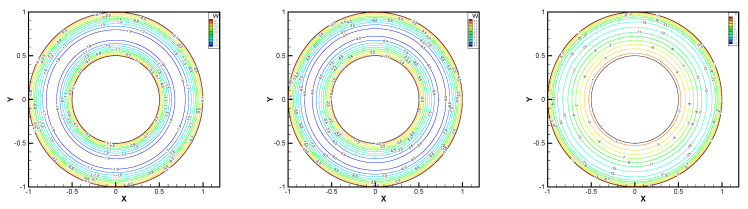
$\omega_{r}=100,500,1000$, angular velocity for the local Gauss integration method.

**Table 1:** Typical structured mesh with $\varepsilon =O(h^{\frac{1}{2}})$ for the penalty method.

$\frac{1}{h}$	CPU	$\frac{∥u\;-\;u_{\epsilon h}{∥}_{1}}{{∥u∥}_{1}}$	$u_{H1}$-Rate	$\frac{∥\omega \;-\;\omega_{\epsilon h}{∥}_{1}}{{∥\omega ∥}_{1}}$	$\omega_{H1}$-Rate	$\frac{∥p\;-\;p_{\epsilon h}{∥}_{0}}{{∥p∥}_{0}}$	$p_{L2}$-Rate
12	0.071		$1.512\times 10^{0}$		$8.828\times 10^{-1}$		$9.606\times 10^{-2}$
24	0.320	0.4089	$1.139\times 10^{0}$	0.4948	$5.615\times 10^{-1}$	0.6529	$6.817\times 10^{-2}$
36	0.846	0.4332	$9.555\times 10^{-1}$	0.4873	$4.405\times 10^{-1}$	0.5985	$5.595\times 10^{-2}$
48	1.755	0.4440	$8.409\times 10^{-1}$	0.4841	$3.740\times 10^{-1}$	0.5683	$4.867\times 10^{-2}$
60	3.185	0.4505	$7.605\times 10^{-1}$	0.4826	$3.309\times 10^{-1}$	0.5497	$4.370\times 10^{-2}$
72	5.332	0.4551	$6.999\times 10^{-1}$	0.4819	$3.000\times 10^{-1}$	0.5373	$4.003\times 10^{-2}$

**Table 2:** Typical structured mesh with $\varepsilon =10^{-6}$ for the penalty method.

$\frac{1}{h}$	CPU	$\frac{∥u\;-\;u_{\epsilon h}{∥}_{0}}{{∥u∥}_{0}}$	$u_{L2}$-Rate	$\frac{∥u\;-\;u_{\epsilon h}{∥}_{1}}{{∥u∥}_{1}}$	$u_{H1}$-Rate	$\frac{∥\omega \;-\;\omega_{\epsilon h}{∥}_{0}}{{∥\omega ∥}_{0}}$	$\omega_{L2}$-Rate	$\frac{∥p\;-\;p_{\epsilon h}{∥}_{0}}{{∥p∥}_{0}}$	$p_{L2}$-Rate
12	0.046	$8.656\times 10^{-2}$		$2.848\times 10^{-1}$		$3.419\times 10^{-2}$		$1.912\times 10^{-1}$	
24	0.225	$2.137\times 10^{-2}$	2.018	$1.412\times 10^{-1}$	1.012	$8.601\times 10^{-3}$	1.991	$1.012\times 10^{-1}$	0.917
36	0.668	$9.447\times 10^{-3}$	2.014	$9.386\times 10^{-2}$	1.008	$3.825\times 10^{-3}$	1.999	$7.059\times 10^{-2}$	0.890
48	1.47	$5.298\times 10^{-3}$	2.010	$7.027\times 10^{-2}$	1.006	$2.152\times 10^{-3}$	2.000	$5.549\times 10^{-2}$	0.836
60	2.806	$3.385\times 10^{-3}$	2.008	$5.615\times 10^{-2}$	1.005	$1.377\times 10^{-3}$	2.001	$4.671\times 10^{-2}$	0.772
72	4.965	$2.348\times 10^{-3}$	2.005	$4.675\times 10^{-2}$	1.005	$9.560\times 10^{-4}$	2.001	$4.109\times 10^{-2}$	0.704

**Table 3:** Typical structured mesh for the regular method.

$\frac{1}{h}$	CPU	$\frac{∥u\;-\;u_{\mathrm{Rh}}{∥}_{0}}{{∥u∥}_{0}}$	$u_{L2}$-Rate	$\frac{∥u\;-\;u_{Rh}{∥}_{1}}{{∥u∥}_{1}}$	$u_{H1}$-Rate	$\frac{∥\omega \;-\;\omega_{Rh}{∥}_{0}}{{∥\omega ∥}_{0}}$	$\omega_{L2}$-Rate	$\frac{∥p\;-\;p_{Rh}{∥}_{0}}{{∥p∥}_{0}}$	$p_{L2}$-Rate
12	0.053	$1.641\times 10^{-1}$		$4.454\times 10^{-1}$		$3.225\times 10^{-2}$		$3.045\times 10^{-2}$	
24	0.281	$4.069\times 10^{-2}$	2.012	$1.800\times 10^{-1}$	1.307	$8.121\times 10^{-3}$	1.990	$9.207\times 10^{-3}$	1.726
36	0.734	$1.797\times 10^{-2}$	2.016	$1.077\times 10^{-1}$	1.268	$3.613\times 10^{-3}$	1.998	$4.675\times 10^{-3}$	1.671
48	1.517	$1.001\times 10^{-2}$	2.013	$7.565\times 10^{-2}$	1.226	$2.033\times 10^{-3}$	1.999	$2.916\times 10^{-3}$	1.641
60	2.768	$6.430\times 10^{-3}$	2.011	$5.794\times 10^{-2}$	1.195	$1.301\times 10^{-3}$	2.000	$2.032\times 10^{-3}$	1.619
72	4.65	$4.457\times 10^{-3}$	2.010	$4.680\times 10^{-2}$	1.171	$9.034\times 10^{-4}$	2.000	$1.517\times 10^{-3}$	1.604

**Table 4:** Typical structured mesh for the multiscale enrichment method.

$\frac{1}{h}$	CPU	$\frac{∥u\;-\;u_{\mathrm{Mh}}{∥}_{0}}{{∥u∥}_{0}}$	$u_{L2}$-Rate	$\frac{∥u\;-\;u_{Mh}{∥}_{1}}{{∥u∥}_{1}}$	$u_{H1}$-Rate	$\frac{∥\omega \;-\;\omega_{\mathrm{Mh}}{∥}_{0}}{{∥\omega ∥}_{0}}$	$\omega_{L2}$-Rate	$\frac{∥p\;-\;p_{\mathrm{Mh}}{∥}_{0}}{{∥p∥}_{0}}$	$p_{L2}$-Rate
12	0.1	$1.723\times 10^{-1}$		$4.387\times 10^{-1}$		$3.394\times 10^{-2}$		$3.116\times 10^{-2}$	
24	0.529	$4.429\times 10^{-2}$	1.960	$1.777\times 10^{-1}$	1.304	$8.847\times 10^{-3}$	1.940	$9.466\times 10^{-3}$	1.719
36	1.446	$2.021\times 10^{-2}$	1.936	$1.067\times 10^{-1}$	1.259	$4.063\times 10^{-3}$	1.919	$4.814\times 10^{-3}$	1.668
48	3.112	$1.170\times 10^{-2}$	1.900	$7.520\times 10^{-2}$	1.216	$2.361\times 10^{-3}$	1.888	$3.005\times 10^{-3}$	1.638
60	5.755	$7.718\times 10^{-3}$	1.863	$5.775\times 10^{-2}$	1.183	$1.561\times 10^{-3}$	1.853	$2.095\times 10^{-3}$	1.617
72	9.652	$5.533\times 10^{-3}$	1.825	$4.676\times 10^{-2}$	1.158	$1.121\times 10^{-3}$	1.817	$1.564\times 10^{-3}$	1.602

**Table 5:** Typical structured mesh for the local Gauss integration method.

$\frac{1}{h}$	CPU	$\frac{∥u\;-\;u_{\mathrm{Gh}}{∥}_{0}}{{∥u∥}_{0}}$	$u_{L2}$-Rate	$\frac{∥u\;-\;u_{Gh}{∥}_{1}}{{∥u∥}_{1}}$	$u_{H1}$-Rate	$\frac{∥\omega \;-\;\omega_{\mathrm{Gh}}{∥}_{0}}{{∥\omega ∥}_{0}}$	$\omega_{L2}$-Rate	$\frac{∥p\;-\;p_{\mathrm{Gh}}{∥}_{0}}{{∥p∥}_{0}}$	$p_{L2}$-Rate
12	0.054	$2.127\times 10^{-1}$		$4.612\times 10^{-1}$		$3.313\times 10^{-2}$		$2.909\times 10^{-2}$	
24	0.256	$5.273\times 10^{-2}$	2.012	$1.753\times 10^{-1}$	1.396	$8.336\times 10^{-3}$	1.991	$8.306\times 10^{-3}$	1.809
36	0.682	$2.335\times 10^{-2}$	2.009	$1.036\times 10^{-1}$	1.297	$3.707\times 10^{-3}$	1.999	$4.103\times 10^{-3}$	1.739
48	1.357	$1.311\times 10^{-2}$	2.006	$7.269\times 10^{-2}$	1.232	$2.085\times 10^{-3}$	2.000	$2.561\times 10^{-3}$	1.700
60	2.403	$8.385\times 10^{-3}$	2.003	$5.575\times 10^{-2}$	1.189	$1.334\times 10^{-3}$	2.001	$1.733\times 10^{-3}$	1.672
72	3.938	$5.823\times 10^{-3}$	2.000	$4.513\times 10^{-2}$	1.159	$9.264\times 10^{-4}$	2.001	$1.282\times 10^{-3}$	1.652

**Table 6:** Unstructured mesh for the penalty method.

$\frac{1}{h}$	$\frac{∥u\;-\;u_{\epsilon h}{∥}_{0}}{{∥u∥}_{0}}$	$u_{L2}$-Rate	$\frac{∥u\;-\;u_{\epsilon h}{∥}_{1}}{{∥u∥}_{1}}$	$u_{H1}$-Rate	$\frac{∥\omega \;-\;\omega_{\epsilon h}{∥}_{0}}{{∥\omega ∥}_{0}}$	$\omega_{L2}$-Rate	$\frac{∥p\;-\;p_{\epsilon h}{∥}_{0}}{{∥p∥}_{0}}$	$p_{L2}$-Rate
0.270282	$1.0559\times 10^{0}$		$1.0587\times 10^{-0}$		$1.1639\times 10^{-1}$		$9.5639\times 10^{-2}$	
0.14633	$1.7658\times 10^{-1}$	2.91	$6.6212\times 10^{-1}$	0.77	$2.6803\times 10^{-2}$	2.39	$9.9276\times 10^{-3}$	3.69
0.073319	$2.1140\times 10^{-2}$	3.07	$1.7191\times 10^{-1}$	1.95	$6.6651\times 10^{-3}$	2.01	$2.5280\times 10^{-3}$	1.98
0.0368164	$3.3790\times 10^{-3}$	2.66	$5.6879\times 10^{-2}$	1.61	$1.6494\times 10^{-3}$	2.03	$7.4832\times 10^{-4}$	1.77
0.0188245	$7.3197\times 10^{-4}$	2.28	$2.5374\times 10^{-2}$	1.20	$4.1029\times 10^{-4}$	2.07	$1.8817\times 10^{-4}$	2.06

**Table 7:** Unstructured mesh for the regular method.

$\frac{1}{h}$	$\frac{∥u\;-\;u_{\mathrm{Rh}}{∥}_{0}}{{∥u∥}_{0}}$	$u_{L2}$-Rate	$\frac{∥u\;-\;u_{Rh}{∥}_{1}}{{∥u∥}_{1}}$	$u_{H1}$-Rate	$\frac{∥\omega \;-\;\omega_{Rh}{∥}_{0}}{{∥\omega ∥}_{0}}$	$\omega_{L2}$-Rate	$\frac{∥p\;-\;p_{Rh}{∥}_{0}}{{∥p∥}_{0}}$	$p_{L2}$-Rate
0.270282	$1.8999\times 10^{-1}$		$4.0094\times 10^{-1}$		$1.4119\times 10^{-1}$		$6.1590\times 10^{0}$	
0.14633	$5.0548\times 10^{-2}$	2.16	$1.9939\times 10^{-1}$	1.14	$3.3420\times 10^{-2}$	2.35	$2.0315\times 10^{0}$	1.81
0.073319	$1.2644\times 10^{-2}$	2.01	$9.8450\times 10^{-2}$	1.02	$8.3367\times 10^{-3}$	2.01	$5.4865\times 10^{-1}$	1.89
0.0368164	$3.1384\times 10^{-3}$	2.02	$4.8764\times 10^{-2}$	1.02	$2.0596\times 10^{-3}$	2.03	$1.5511\times 10^{-1}$	1.83
0.0188245	$7.9530\times 10^{-3}$	2.05	$2.4324\times 10^{-2}$	1.04	$5.1178\times 10^{-4}$	2.08	$4.8886\times 10^{-1}$	1.72

**Table 8:** Unstructured mesh for the multiscale enrichment method.

$\frac{1}{h}$	$\frac{∥u\;-\;u_{\mathrm{Mh}}{∥}_{0}}{{∥u∥}_{0}}$	$u_{L2}$-Rate	$\frac{∥u\;-\;u_{Mh}{∥}_{1}}{{∥u∥}_{1}}$	$u_{H1}$-Rate	$\frac{∥\omega \;-\;\omega_{\mathrm{Mh}}{∥}_{0}}{{∥\omega ∥}_{0}}$	$\omega_{L2}$-Rate	$\frac{∥p\;-\;p_{\mathrm{Mh}}{∥}_{0}}{{∥p∥}_{0}}$	$p_{L2}$-Rate
0.270282	$5.3045\times 10^{-1}$		$5.7977\times 10^{0}$		$1.9495\times 10^{-1}$		$3.9490\times 10^{0}$	
0.14633	$1.6679\times 10^{-1}$	1.89	$2.8543\times 10^{0}$	1.15	$5.3267\times 10^{-2}$	2.11	$1.6592\times 10^{0}$	1.41
0.073319	$4.9313\times 10^{-2}$	1.76	$1.3982\times 10^{0}$	1.03	$1.4854\times 10^{-2}$	1.85	$4.9800\times 10^{-1}$	1.74
0.0368164	$1.5745\times 10^{-2}$	1.66	$6.9553\times 10^{-1}$	1.01	$4.3683\times 10^{-3}$	1.78	$1.6378\times 10^{-1}$	1.61
0.0188245	$5.8796\times 10^{-3}$	1.47	$3.5345\times 10^{-1}$	1.01	$1.4678\times 10^{-3}$	1.63	$6.3343\times 10^{-2}$	1.42

**Table 9:** Unstructured mesh for the local Gauss integration method.

$\frac{1}{h}$	$\frac{∥u\;-\;u_{\mathrm{Gh}}{∥}_{0}}{{∥u∥}_{0}}$	$u_{L2}$-Rate	$\frac{∥u\;-\;u_{Gh}{∥}_{1}}{{∥u∥}_{1}}$	$u_{H1}$-Rate	$\frac{∥\omega \;-\;\omega_{\mathrm{Gh}}{∥}_{0}}{{∥\omega ∥}_{0}}$	$\omega_{L2}$-Rate	$\frac{∥p\;-\;p_{\mathrm{Gh}}{∥}_{0}}{{∥p∥}_{0}}$	$p_{L2}$-Rate
0.270282	$1.8272\times 10^{-1}$		$3.9585\times 10^{-1}$		$1.4450\times 10^{-1}$		$4.6246\times 10^{0}$	
0.14633	$4.5578\times 10^{-2}$	2.26	$1.9863\times 10^{-1}$	1.12	$3.4643\times 10^{-2}$	2.33	$1.9980\times 10^{0}$	1.37
0.073319	$1.1183\times 10^{-2}$	2.03	$9.8443\times 10^{-2}$	1.02	$8.6578\times 10^{-3}$	2.01	$6.1863\times 10^{-1}$	1.70
0.0368164	$2.7425\times 10^{-3}$	2.04	$4.8775\times 10^{-2}$	1.02	$2.1423\times 10^{-2}$	2.03	$1.9223\times 10^{-1}$	1.70
0.0188245	$6.8186\times 10^{-4}$	2.07	$2.4329\times 10^{-2}$	1.04	$5.3335\times 10^{-4}$	2.07	$6.5361\times 10^{-2}$	1.61

## Data Availability

Not applicable.

## References

[1] Eringen A.C. (1966). Theory of micropolar fluids. J. Math. Mech..

[2] Girault V., Raviart P.A. (1986). Finite Element Methods for Navier Stokes Equations: Theory and Algorithms.

[3] Brezzi F., Fortin M. (1991). Mixed and Hybrid Finite Element Methods.

[4] Douglas J., Wang J.P. (1989). An absolutely stabilized finite element method for the Stokes problem. Math. Comput..

[5] Bochev P., Dohrmann C., Gunzburger M. (2006). Stabilization of low-order mixed finite elements for the Stokes equations. SIAM J. Numer. Anal..

[6] Li J., He Y., Chen Z. (2009). Performance of several stabilized finite element methods for the Stokes equations based on the lowest equal-order pairs. Computing.

[7] Kechar N., Silvester D. (1992). Analysis of a locally stabilized mixed finite element method for the Stokes problem. Math. Comput..

[8] Carey G.F., Krishnan R. (1982). Penalty approximation of stokes flow. Comput. Methods Appl. Mech. Eng..

[9] Barrenechea G., Valentin F. (2002). An unusual stabilized finite element method for a generalized Stokes problem. Numer. Math..

[10] Li J., He Y. (2008). A stabilized finite element method based on two local Gauss integrations for the Stokes equations. J. Comput. Appl. Math..

[11] Araya R., Barrenechea G., Valentin F. (2006). Stabilized finite element methods based on multiscale enrichment for the Stokes problem. SIAM J. Numer. Anal..

[12] Franca L., Madureira A., Tobiska L., Valentin F. (2005). Convergence analysis of a multiscale finite element method for singularly perturbed problems. Multiscale Model. Simul..

[13] Franca L., Hughes T.J.R., Stenberg R., Gunzburger M., Nicolaides R. (1993). Stabilized finite element methods. Incompressible Computational Fluid Dynamics.

[14] Baiocchi C., Brezzi F., Franca L.P. (1993). Virtual bubbles and Galerkin-least-squares type methods. Comput. Methods Appl. Mech. Eng..

[15] Yang L., Badia S., Codina R. (2016). A pseudo-compressible variational multiscale solver for turbulent incompressible flows. Comput. Mech..

[16] Temam R. (2001). Navier-Stokes Equations-Theory and Numerical Analysis.

[17] Heywood J.G. (1980). The Navier-Stokes Equations: On the Existence, Regularity and Decay of Solutions. Indiana Univ. J. Math..

[18] Ciarlet P.G. (1978). The Finite Element Method for Elliptic Problems.

[19] Fortin M. (1977). An analysis of the convergence of mixed finite element methods. RAIRO Anal. Numer..

[20] Ern A., Guermond J.L. (2004). Theory and Practice of Finite Elements.

[21] Clément P. (1975). Approximation by finite element functions using local regularization. RAIRO Anal. Numer..

